# Graded mesh B-spline collocation method for two parameters singularly perturbed boundary value problems

**DOI:** 10.1016/j.mex.2023.102336

**Published:** 2023-08-25

**Authors:** Fellek Sabir Andisso, Gemechis File Duressa

**Affiliations:** a Department of Mathematics, Arba Minch University, Arba Minc, 21, Ethiopia; b Department of Mathematics, Jimma University, Jimma, 378, Ethiopia

**Keywords:** Layer adapted, Singularly perturbed, B-Spline collocation, Graded mesh, Boundary layers, parameters uniform convergent, Cubic B-spline collocation method

## Abstract

The solutions of two parameters singularly perturbed boundary value problems typically exhibit two boundary layers. Because of the presence of these layers standard numerical methods fail to give accurate approximations. This paper introduces a numerical treatment of a class of two parameters singularly perturbed boundary value problems whose solution exhibits boundary layer phenomena. A graded mesh is considered to resolve the boundary layers and collocation method with cubic B-splines on the graded mesh is proposed and analyzed. The proposed method leads to a tri-diagonal linear system of equations. The stability and parameters uniform convergence of the present method are examined. To verify the theoretical estimates and efficiency of the method several known test problems in the literature are considered. Comparisons to some existing results are made to show the better efficiency of the proposed method. Summing up:

• The present method is found to be stable and parameters uniform convergent and the numerical results support the theoretical findings.
• Experimental results show that the present method approximates the solution very well and has a rate of convergence of order two in the maximum norm.
• Experimental results show that cubic B-spline collocation method on graded mesh is more efficient than cubic B-spline collocation method on Shishkin mesh and some other existing methods in the literature.

## Graphical abstract

**Figure:**
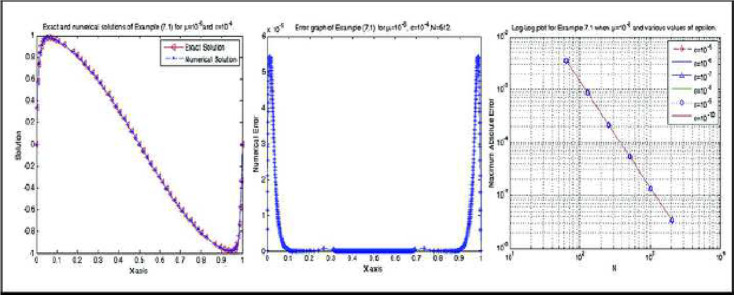

Specifications table

**Table:** 

Subject Area:	Mathematics
More Specific Subject Area:	Numerical Analysis
Method name:	Cubic B-spline collocation method
Name and reference of original method:	M. K. Kadalbajoo, A. S. Yadaw, B-spline collocation method for a two-parameter singularly perturbed convection-diffusion boundary value problems, Applied Mathematics and Computation 201 (1–2) (2008) 504–513. doi:https://doi.org/10.1016/j.amc.2007.12.038.
Resource availability:	Matlab software package

## Introduction

Differential equations having small perturbation parameters multiplying the highest and higher derivative terms are called singularly perturbed problems (SPPs). The solutions of these types of problems exhibit multi scale characters. That is, there are a narrow region called boundary layer in which their solution changes rapidly and the outer region where solution changes smoothly. Classical numerical methods are known to be inadequate to solve such multi scale problems unless extremely fine meshes are used. However, this fine uniform mesh requirement by most of the classical methods for stability in the singularly perturbed case leads significantly large algebraic system. Hence this incorporates the massive computational cost.Therefore, design of robust layer adapted meshes for such problems is an important task. In this paper the following problem is considered:

(1) $$Lu\left(x\right)=-\varepsilon u^{\prime \prime }\left(x\right)+\mu a\left(x\right)u^{\prime }\left(x\right)+b\left(x\right)u\left(x\right)=f\left(x\right),$$

with boundary conditions

(2) $$u\left(0\right)=\alpha_{0},u\left(1\right)=\alpha_{1},$$

where $\alpha_{0},\;\alpha_{1}$ are given scalars and the two small perturbation parameters 0<ϵ,μ≪1 are such that $\frac{\mu^{2}}{\varepsilon }\to 0$ as ε→0 ($\mu^{2}\ll \epsilon$) or $\frac{\varepsilon }{\mu^{2}}\to 0$ as μ→0 ($\varepsilon \ll \mu^{2}$). The functions a(x), b(x), and f(x) are assumed to be sufficiently smooth functions and satisfying

(3) $$a\left(x\right)\geq \alpha >0,\;b\left(x\right)\geq \beta >0,\forall x\in \bar{\Omega }=\left[0,1\right].$$

Such types of problems are called two parameters singularly perturbed boundary value problems (TPSPBVPs). The applications of such types of problems appear in transport phenomena arising in chemistry and biology [1], in lubrication theory [2], in chemical reactor theory [3], and so on.

When μ=0, the problem (1)-(2) reduces to the well-studied reaction diffusion problem of single parameter singularly perturbed boundary value problem (SPSPBVP) while for μ=1 it becomes the convection diffusion problem. For the problem classes of these two special cases, there is a huge number of research papers devoted to their numerical solving, however there are only few number of research papers for the purely two parameters case 0<ε,μ≪1. For problem classes of case μ=0 (reaction diffusion problem)for instance research papers like  [4], [5], [6], [7], [8] and for μ=1 (convection diffusion problem) papers like [9], [10], [11], [12] are available in the literature.

The solution of problem (1)-(2) exhibits narrow regions called inner(boundary layer) regions where it behaves irregularly and changes rapidly and the outer region where it behaves regularly and changes slowly. Because of the presence of the boundary layers the classical numerical methods are known to be unsuitable & fail to give accurate results when the perturbation parameters are small unless extremely fine meshes are used. But this fine mesh requirement by the classical methods leads to a very large algebraic system which incorporates high computational cost. Therefore it is important to develop suitable parameters uniform layer adapted numerical methods to get approximate solutions of such problems. Parameters uniform numerical methods are methods which satisfy error bounds of the form

(4) $$∥u_{\varepsilon ,\mu }-U^{N}∥\leq c\vartheta \left(N\right),\vartheta \left(N\right)\to 0\;as\;N\to \infty ,$$

where $u_{\varepsilon ,\mu }$ is exact solution of the continuous problem, $U^{N}$ is numerical approximation of the exact solution $u_{\varepsilon ,\mu }$, ∥·∥ is the maximum pointwise norm, N is the number of mesh intervals used and c is a positive constant independent of N and the parameters. A numerical method is said to be parameters uniformly convergent of order p if

(5) $$∥u_{\varepsilon ,\mu }-U^{N}∥\leq cN^{-p},p>0.$$

For further details, the reader is referred to the books and research papers available in the literature. Among the books  [13], [14], [15], [16], [17] are mentioned. Some of the research papers available in the already existing literature are expressed as follows. O’Malley in [18] studied the nature of the two parameter problem asymptotically and examined that the solution behavior depends on the ratios $\frac{\varepsilon }{\mu^{2}}$ as μ→0 and $\frac{\mu^{2}}{\varepsilon }$ as ε→0. Roos & Uzelac in [19] constructed a streamline diffusion finite element method on Shishkin mesh and shown that the method is almost second order parameters uniformly convergent. The authors in [20] proposed upwind difference method on Shishkin-Bakhvalov mesh to solve a TPSPBVP and found the method to be first order parameters uniform convergent. Linß & Roos in [21] used a simple upwind difference scheme on Shishkin mesh to solve a TPSPBVP and found the method to be parameters uniformly convergent of almost order one. The authors in [22] have proposed B-spline collocation method on Shishkin mesh & shown that it has a second order parameters uniform convergence. The Ritz-Galerkin method has been proposed by Kadalbajoo et.al in [23]. These authors have used a piecewise uniform Shishkin mesh to resolve the boundary layers and the method has been found to be almost second order parameters uniform convergent.

The authors in [24] presented a comparative study of fitted mesh finite difference, Ritz-Galerkin finite element and B-spline collocation methods on Shishkin mesh. They have shown that B-spline collocation method has second order parameters uniform convergence, the Ritz-Galerkin finite element method has almost second order parameters uniform convergence and the finite difference method has almost first order parameters uniform convergence. In [25] a semi-linear TPSPBVPs is solved using exponential spline on a Shishkin mesh and the method is shown to be parameters uniformly convergent. The authors in [26] developed a quadratic B-spline collocation method for TPSPBVPs on exponentially graded mesh and they have shown the method to be parameters uniformly convergent of order two.

Besides the fact that there are fewer number of research papers for TPSPBVPs as compared to the number of research papers on SPSPBVPs, most of the research papers on TPSPBVPs are on Shishkin meshes. As far as our knowledge of the literature the number of research papers available on graded meshes is very few as compared to those on Shishkin mesh. Moreover, for some methods like finite difference method on Shishkin mesh it is known that rate of convergence is deteriorated by the presence of logarithmic factor whereas graded mesh methods which are without logarithmic factors give better rate of convergence. Hence the aim of this paper is to develop and analyze a graded mesh cubic B-spline collocation method using a spline interpolant which satisfies the same boundary conditions for approximating solutions of TPSPBVPs. Experimental results in this paper shown that cubic B-spline collocation method on graded mesh (present method) is better than cubic B-spline collocation method on Shishkin method (existing method). The flow chart for the work is given in the appendix.

## Continuous problem solution properties

In this section bounds on the solution and its derivatives of problem (1)-(2) which will be used in error analysis will be established. Characteristic equation of the homogeneous equation corresponding to the differential equation in (1) is

(6) $$-\varepsilon \lambda^{2}\left(x\right)+\mu a\left(x\right)\lambda \left(x\right)+b\left(x\right)=0.$$

The solutions of Eq. (6) become

(7) $$\lambda_{0}\left(x\right)=\frac{\mu a\left(x\right)-\sqrt{\mu^{2}a{\left(x\right)}^{2}+4\varepsilon b\left(x\right)}}{2\varepsilon },\lambda_{1}\left(x\right)=\frac{\mu a\left(x\right)+\sqrt{\mu^{2}a{\left(x\right)}^{2}+4\varepsilon b\left(x\right)}}{2\varepsilon }.$$

These two real solutions $\lambda_{0}\left(x\right)<0$ & $\lambda_{1}\left(x\right)>0$ describe the boundary layers at x=0 & x=1 respectively. Define

(8) $$v_{0}=-\max_{0\leq x\leq 1}\lambda_{0}\left(x\right)\;\text{and}\;v_{1}=\min_{0\leq x\leq 1}\lambda_{1}\left(x\right)$$

Recall that the solution behavior of problem (1)-(2) depends on the ratios $\frac{\mu^{2}}{\varepsilon }\to 0\;\text{as}\;\varepsilon \to 0$ and $\frac{\varepsilon }{\mu^{2}}\to 0\;\text{as}\;\mu \to 0$.

case (1): $\frac{\mu^{2}}{\varepsilon }\to 0\;\text{as}\;\varepsilon \to 0$.
  In this case one can easily verify that $v_{0}=v_{1}=\sqrt{\frac{\beta }{\varepsilon }}$, which gives:
  (9) $$v_{0}=v_{1}\approx \varepsilon^{-\frac{1}{2}}$$
  Hence behavior of solution in this case is similar to that of the reaction diffusion problem (μ=0) and exhibits boundary layers at x=0 & x=1 with boundary widths of $O(\varepsilon^{-\frac{1}{2}})$ [27], [28] for each layer.
case (2): $\frac{\varepsilon }{\mu^{2}}\to 0\;\text{as}\;\mu \to 0$.
  For this case it is possible to show that
  (10) $$v_{0}\approx \mu^{-1},\;\text{and}\;v_{1}\approx \frac{\mu }{\varepsilon }.$$
  Since the parameters ε and μ are non-zero small numbers and using Eq. (10) it can be observed that $v_{1}=\frac{\mu }{\varepsilon }=\frac{\mu }{\varepsilon }\mu \mu^{-1}=\frac{\mu^{2}}{\varepsilon }\mu^{-1}=\frac{\mu^{2}}{\varepsilon }v_{0}$ implies:
  (11) $$v_{0}\ll v_{1}\;\text{since}\;\frac{\mu^{2}}{\varepsilon }=\frac{1}{\left(\frac{\varepsilon }{\mu^{2}}\right)}\to \infty \;\text{as}\;\mu \to 0.$$
  Hence when $\frac{\varepsilon }{\mu^{2}}\to 0\;\text{as}\;\varepsilon \to 0$, the layer at x=1 is stronger than the layer at x=0 and the boundary layer widths are $O(\mu^{-1})$ and $O(\frac{\mu }{\varepsilon })$ at x=0 and x=1 respectively [27], [28].

Remark 1 For case (2) when a(x)<0, a strong layer at x=0 exists when compared to the layer at x=1.(It means when a(x)<0, $v_{1}\ll v_{0}$).

Moreover for both case (1) and case (2) it can clearly be shown that

(12) $$1\ll v_{0}$$

The operator L in Eq. (1) satisfies the following maximum principle.

Lemma 1 *Assume*u(0)≥0*,*u(1)≥0*and*Lu≥0*on*Ω=(0,1)*. Then,*$u\geq 0\;on\;\bar{\Omega }=\left[0,1\right]$*.*

See [23] for proof.

The following stability estimate is obtained.

Lemma 2 *The solution*u(x)*of problem*(1)*-*(2)*satisfies*

$${∥u∥}_{\left[0,1\right]}\leq \max\left(|\alpha_{0}\left|,\right|\alpha_{1}|\right)+\frac{1}{\beta }∥f{∥}_{\left[0,1\right]}.$$

Proof Define the barrier functions: $\Pi^{\pm }\left(x\right)=\max(|\alpha_{0}\left|,\right|\alpha_{1}\left|\right)+\frac{1}{\beta }{∥f∥}_{\left[0,1\right]}\pm u\left(x\right).$ Clearly it holds that $\Pi^{\pm }\left(0\right)\geq 0$ and $\Pi^{\pm }\left(1\right)\geq 0$. For every x in (0,1) it can be obtained that:

$$\begin{array}{cc} L\Pi^{\pm }\left(x\right) & =-\epsilon {\left[\Pi^{\pm }\left(x\right)\right]}^{\prime \prime }+\mu a\left(x\right){\left[\Pi^{\pm }\left(x\right)\right]}^{\prime }+b\left(x\right)\left[\Pi^{\pm }\left(x\right)\right]\\ & =\frac{b(x)}{\beta }{∥f∥}_{\left[0,1\right]}\pm f\left(x\right)+b\left(x\right)\max(|\alpha_{0}\left|,\right|\alpha_{1}\left|\right) \end{array}$$

This yields $L\Pi^{\pm }\left(x\right)\geq {∥f∥}_{\left[0,1\right]}\pm f\left(x\right)+b\left(x\right)\max(|\alpha_{0}\left|,\right|\alpha_{1}\left|\right)\geq 0,\;\text{since}\;\frac{b(x)}{\beta }\geq 1.$

Hence from maximum principle it follows that $\Pi^{\pm }\left(x\right)\geq 0,\;\forall \;x\in \bar{\Omega }=\left[0,1\right]$, which implies:

$$∥u∥\leq \max(|\alpha_{0}\left|,\right|\alpha_{1}\left|\right)+\frac{1}{\beta }{∥f∥}_{\left[0,1\right]},\;\forall x\in \bar{\Omega }=\left[0,1\right]$$

completing proof of the lemma. □

Theorem 1 *Let*$a,b,f\in C{}^{q}\left[0,1\right]$*for some*q≥1*and let*p,r∈(0,1)*be chosen arbitrarily**[21**,**29]**. Assume*$q∥a^{\prime }∥\mu \leq \beta r\left(1-p\right)$*, then it results to*

(13) $$|u^{\left(k\right)}\left(x\right)|\leq C\left(1+v_{0}^{k}e^{-pv_{0}x}+v_{1}^{k}e^{-pv_{1}\left(1-x\right)}\right),\;x\in \left(0,1\right),\;0\leq k\leq q.$$

*Furthermore, the solution u to the problem*(1)*-*(2)*can be decomposed as*$u\left(x\right)=v\left(x\right)+w_{L}\left(x\right)+w_{R}\left(x\right)$*and for*k=0,1,⋯,q*, it holds*

(14) $$∥v^{\left(k\right)}∥\leq C,x\in \left(0,1\right),$$

(15) $$|w_{L}^{\left(k\right)}\left(x\right)|\leq Cv_{0}^{k}e^{-pv_{0}x},x\in \left(0,1\right),$$

(16) $$|w_{R}^{\left(k\right)}\left(x\right)|\leq Cv_{1}^{k}e^{-pv_{1}\left(1-x\right)},x\in \left(0,1\right),$$

*where*$v,w_{L},\text{and}\;w_{R}$*are regular, left boundary layer and right boundary layer components of solution respectively.*

## Mesh construction

This section presents the graded mesh as constructed in [26]. Define the constants $\varphi_{0}$ and $\varphi_{1}$ by

(17) $$\varphi_{j}=1-e^{\frac{pv_{j}}{2\tau }}\in \left(0,1\right),j=0,1,$$

where p∈(0,1) & τ≥1 is a parameter to be chosen by the user. Let $T=\{t_{i}:t_{i}=\frac{i}{N},i=0,1,\cdots ,N\}$ be a uniform mesh. Let us construct a non-uniform mesh ${\bar{\Omega }}^{N}={\left\{x_{i}\right\}}_{i=0}^{N}$, where N is the number of sub-intervals in [0,1] which is a multiple of 4, N∈N & N≥8. Let the two mesh generating functions $\Phi_{0}$ & $\Phi_{1}$ which are important to resolve the boundary layers be defined by

(18) $$\Phi_{0}\left(t\right)=-\ln\left(1-4\varphi_{0}t\right),t\in \left(0,1\right),$$

(19) $$\Phi_{1}\left(t\right)=-\ln\left[1-4\varphi_{1}\left(1-t\right)\right],t\in \left(0,1\right).$$

Then the mesh points $x_{j},j=0,1,\cdots ,N$ are defined as:

(20) $$x_{j}=\left\{ \begin{array}{c} \frac{\tau }{pv_{0}}\Phi_{0}\left(t_{j}\right),t_{j}=\frac{j}{N},j=0,1,\cdots ,\frac{N}{4}-1,\\ x_{\frac{N}{4}-1}+\frac{x_{\frac{3N}{4}+1}-x_{\frac{N}{4}-1}}{\frac{N}{2}+2}\left(i-\frac{N}{4}+1\right),i=\frac{N}{4},\frac{N}{4}+1,\cdots ,\frac{3N}{4},\\ 1-\frac{\tau }{pv_{1}}\Phi_{1}\left(t_{j}\right),t_{j}=\frac{j}{N},j=\frac{3N}{4}+1,\frac{3N}{4}+2,\cdots ,N, \end{array}\right.$$

Subdividing the interval [0,1] into three subintervals: $\left[0,1\right]=\left[0,x_{\frac{N}{4}-1}\right]\cup \left[x_{\frac{N}{4}-1},x_{\frac{3N}{4}+1}\right]\cup \left[x_{\frac{3N}{4}+1},1\right].$ The mesh points in $\left[x_{\frac{N}{4}-1},x_{\frac{3N}{4}+1}\right]$ are equidistant while they are gradually distributed in $\left[0,x_{\frac{N}{4}-1}\right]$ and $\left[x_{\frac{3N}{4}+1},1\right]$. Let the mesh step size $h_{i}$ be defined by $h_{i}=x_{i}-x_{i-1},i=1,2,\cdots ,N$. The mesh step size $h_{i}$ for i=1,2,⋯,N satisfies the following properties (which will be used in error analysis):

(21) $$h_{i}\leq \left\{ \begin{array}{c} Cv_{0}^{-1},i=1,2,\cdots ,\frac{N}{4}-1,\\ CN^{-1},i=\frac{N}{4},\frac{N}{4}+1,\cdots ,\frac{3N}{4}+1,\\ Cv_{1}^{-1},i=\frac{3N}{4}+2,\frac{3N}{4}+3,\cdots ,N. \end{array}\right.$$

Defining $\psi_{j}=e^{-\Phi_{j}},j=0,1$; it can be proved that the mesh widths in the layer regions satisfy:

(22) $$h_{i}\leq \left\{ \begin{array}{c} Cv_{0}^{-1}N^{-1}e^{\frac{pv_{0}x_{i}}{\tau }},i=1,2,\cdots ,\frac{N}{4}-1,\\ Cv_{1}^{-1}N^{-1}e^{\frac{pv_{1}\left(1-x_{i-1}\right)}{\tau }},i=\frac{3N}{4}+2,\frac{3N}{4}+3,\cdots ,N. \end{array}\right.$$

## Cubic B-splines on non-uniform mesh

**B-splines of degree 0**

The B-splines of degree 0 denoted by $B_{i}^{0}$ are defined by

(23) $$B_{i}^{0}\left(x\right)=\left\{ \begin{array}{cc} 1 & \text{if}\;x_{i}\leq x\leq x_{i+1}\\ 0 & \;\text{otherwise}. \end{array}\right.$$

B-splines of degree k as in [30] are defined by the recurrence relation

(24) $$B_{i}^{k}\left(x\right)=\left(\frac{x-x_{i}}{x_{i+k}-x_{i}}\right)B_{i}^{k-1}\left(x\right)+\left(\frac{x_{i+k+1}-x}{x_{i+k+1}-x_{i+1}}\right)B_{i+1}^{k-1}\left(x\right),k\geq 1$$

(25) $$\text{Let}\;v_{i}^{k}\left(x\right)=\frac{x-x_{i}}{x_{i+k}-x_{i}}$$

Then the recurrence relation (24) above can be re-written in the form:

(26) $$B_{i}^{k}\left(x\right)=v_{i}^{k}B_{i}^{k-1}+\left(1-v_{i+1}^{k}\right)B_{i+1}^{k-1}$$

Since $B_{i}^{0}$ is a piecewise polynomial of degree zero and since $v_{i}^{k}$ is linear, $B_{i}^{1}$ is a piecewise polynomial of degree less than or equal to one. Similarly through same reasoning, $B_{i}^{k}$ is a piecewise polynomial of degree less than or equal to k.

Lemma 3 *If*k≥1*and*$x\notin (x_{i},x_{i+k+1})$*, then*$B_{i}^{k}\left(x\right)=0$*[30]**.*

This means the support for $B_{i}^{k}\left(x\right)$ is $\left(x_{i},x_{i+k+1}\right)$.

Lemma 4 *Let*k≥0*. If*$x\in (x_{i},x_{i+k+1})$*, then*$B_{i}^{k}\left(x\right)>0$*[30]**.*

This implies that B-splines are positive and never negative.

Lemma 5 *For all k,*$\sum_{i=-\infty }^{\infty }B_{i}^{k}\left(x\right)=1$*[30]**.*

For cubic B-splines k=3, the recursive formula (24) above becomes:

(27) $$B_{i}^{3}\left(x\right)=\left(\frac{x-x_{i}}{x_{i+3}-x_{i}}\right)B_{i}^{2}\left(x\right)+\left(\frac{x_{i+4}-x}{x_{i+4}-x_{i+1}}\right)B_{i+1}^{2}\left(x\right).$$

and the support for $B_{i}^{3}\left(x\right)$ is $\left(x_{i},x_{i+4}\right)$. By recursively using (27) for $B_{i}^{3}$, it is possible to obtain:

(28) $$B_{i}^{3}\left(x\right)=\left\{ \begin{array}{cc} \frac{{\left(x-x_{i-2}\right)}^{3}}{\left(x_{i+1}-x_{i-2}\right)\left(x_{i}-x_{i-2}\right)\left(x_{i-1}-x_{i-2}\right)}, & \text{if}\;x_{i-2}\leq x\leq x_{i-1},\\ \frac{{\left(x-x_{i-2}\right)}^{2}\left(x_{i}-x\right)}{\left(x_{i+1}-x_{i-2}\right)\left(x_{i}-x_{i-2}\right)\left(x_{i}-x_{i-1}\right)}+\frac{\left(x-x_{i-2}\right)\left(x_{i+1}-x\right)\left(x-x_{i-1}\right)}{\left(x_{i+1}-x_{i-2}\right)\left(x_{i+1}-x_{i-1}\right)\left(x_{i}-x_{i-1}\right)}+ & \\ \frac{\left(x_{i+2}-x\right){\left(x-x_{i-1}\right)}^{2}}{\left(x_{i+2}-x_{i-1}\right)\left(x_{i+1}-x_{i-1}\right)\left(x_{i}-x_{i-1}\right)}, & \text{if}\;x_{i-1}\leq x\leq x_{i},\\ \frac{{\left(x_{i+2}-x\right)}^{2}\left(x-x_{i}\right)}{\left(x_{i+2}-x_{i-1}\right)\left(x_{i+2}-x_{i}\right)\left(x_{i+1}-x_{i}\right)}+\frac{\left(x_{i+2}-x\right)\left(x-x_{i-1}\right)\left(x_{i+1}-x\right)}{\left(x_{i+2}-x_{i-1}\right)\left(x_{i+1}-x_{i-1}\right)\left(x_{i+1}-x_{i}\right)}+ & \\ \frac{\left(x-x_{i-2}\right){\left(x_{i+1}-x\right)}^{2}}{\left(x_{i+1}-x_{i-2}\right)\left(x_{i+1}-x_{i-1}\right)\left(x_{i+1}-x_{i}\right)}, & \;\text{if}\;x_{i}\leq x\leq x_{i+1},\\ \frac{{\left(x_{i+2}-x\right)}^{3}}{\left(x_{i+2}-x_{i-1}\right)\left(x_{i+2}-x_{i}\right)\left(x_{i+2}-x_{i+1}\right)}, & \;\text{if}\;x_{i+1}\leq x\leq x_{i+2},\\ 0, & \text{otherwise}. \end{array}\right.$$

Following B-spline values which will be used latter in the next section can be obtained from the above definition of B-splines.

(29) $$B_{0}^{3}\left(x_{0}\right)=\frac{h_{1}+h_{2}}{2h_{1}+h_{2}}.$$

(30) $$B_{N}^{3}\left(x_{N}\right)=\frac{h_{N-1}+h_{N}}{h_{N-1}+2h_{N}}.$$

(31) $$B_{-1}^{3}\left(x_{0}\right)=\frac{h_{1}}{2(2h_{1}+h_{2})}.$$

(32) $$B_{N-1}^{3}\left(x_{N}\right)=\frac{h_{N}}{2(h_{N-1}+2h_{N})}.$$

(33) $$B_{1}^{3}\left(x_{0}\right)=\frac{h_{1}}{2(2h_{1}+h_{2})}.$$

(34) $$B_{N+1}^{3}\left(x_{N}\right)=\frac{h_{N}}{2(h_{N-1}+2h_{N})}.$$

## The numerical scheme

In this section, a cubic B-spline collocation method on the graded mesh is derived.

$\Omega^{N}=\left\{0=x_{0}<x_{1}<x_{2}<\cdots <x_{N}=1\right\}$ as described by Eq. (20).

Hereafter $B_{i}\left(x\right)$ is used instead of $B_{i}^{3}\left(x\right)$ as long as there is no ambiguity. The partition $\Omega^{N}$ is extended by introducing 6 extra knots outside the interval [0,1]. Specifically, the mesh points such that $x_{-3}<x_{-2}<x_{-1}<x_{0}=0$ and $1=x_{N}<x_{N+1}<x_{N+2}<x_{N+3}$ are introduced. Then for i=−1,0,1,⋯,N+1, the cubic B-splines $B_{-1},B_{0},B_{1},\cdots ,B_{N+1}$ are defined according to Eq. (28).

An approximate solution U(x) of problem (1)- (2) is sought for and is in the form:

(35) $$U\left(x\right)=\sum_{i=-1}^{N+1}\gamma_{i}B_{i}\left(x\right),$$

where $\gamma_{i}$ are unknown real parameters to be determined. The function U(x) should satisfy the differential equation in Eq. (1) at the mesh points of the partition $\Omega^{N}$ as well as the boundary conditions at $x=x_{0}$ and $x=x_{N}$. Hence, the following hold:

(36) $$LU\left(x_{j}\right)=f\left(x_{j}\right),j=0,1,\cdots ,N,$$

(37) $$U\left(x_{0}\right)=\alpha_{0},U\left(x_{N}\right)=\alpha_{1}.$$

Since only 3 cubic B-splines, namely $B_{j-1},B_{j}\;$& $B_{j+1}$, contribute to the value of U(x) at a particular node $x_{j}$, 0≤j≤N, the following hold:

(38) $$U\left(x_{j}\right)=\gamma_{j-1}B_{j-1}\left(x_{j}\right)+\gamma_{j}B_{j}\left(x_{j}\right)+\gamma_{j+1}B_{j+1}\left(x_{j}\right),j=0,1,2,\cdots ,N.$$

(39) $$U^{\prime }\left(x_{j}\right)=\gamma_{j-1}B_{j-1}^{\prime }\left(x_{j}\right)+\gamma_{j}B_{j}^{\prime }\left(x_{j}\right)+\gamma_{j+1}B_{j+1}^{\prime }\left(x_{j}\right),j=0,1,2,\cdots ,N.$$

(40) $$U^{\prime \prime }\left(x_{j}\right)=\gamma_{j-1}B_{j-1}^{\prime \prime }\left(x_{j}\right)+\gamma_{j}B_{j}^{\prime \prime }\left(x_{j}\right)+\gamma_{j+1}B_{j+1}^{\prime \prime }\left(x_{j}\right),j=0,1,2,\cdots ,N.$$

$LU\left(x_{j}\right)=L\sum_{i=-1}^{N+1}\gamma_{i}B_{i}\left(x_{j}\right)=f\left(x_{j}\right),j=0,1,\cdots ,N.$

$LU\left(x_{j}\right)=-\varepsilon U^{\prime \prime }\left(x_{j}\right)+\mu a_{j}U^{\prime }\left(x_{j}\right)+b_{j}U\left(x_{j}\right)=f\left(x_{j}\right),j=0,1,\cdots ,N.$

$$\begin{array}{cc} & -\varepsilon \left[\gamma_{j-1}B_{j-1}^{\prime \prime }\left(x_{j}\right)+\gamma_{j}B_{j}^{\prime \prime }\left(x_{j}\right)+\gamma_{j+1}B_{j+1}^{\prime \prime }\left(x_{j}\right)\right]+\mu a_{j}\left[\gamma_{j-1}B_{j-1}^{\prime }\left(x_{j}\right)+\gamma_{j}B_{j}^{\prime }\left(x_{j}\right)+\gamma_{j+1}B_{j+1}^{\prime }\left(x_{j}\right)\right]\\ & \;+b_{j}\left[\gamma_{j-1}B_{j-1}\left(x_{j}\right)+\gamma_{j}B_{j}\left(x_{j}\right)+\gamma_{j+1}B_{j+1}\left(x_{j}\right)\right]=f\left(x_{j}\right),j=0,1,\cdots ,N. \end{array}$$

(41) $$\begin{array}{cc} & \left[-\varepsilon B_{j-1}^{\prime \prime }\left(x_{j}\right)+\mu a_{j}B_{j-1}^{\prime }\left(x_{j}\right)+b_{j}B_{j-1}\left(x_{j}\right)\right]\gamma_{j-1}+\left[-\varepsilon B_{j}^{\prime \prime }\left(x_{j}\right)+\mu a_{j}B_{j}^{\prime }\left(x_{j}\right)+b_{j}B_{j}\left(x_{j}\right)\right]\gamma_{j}\\ & \;+\left[-\varepsilon B_{j+1}^{\prime \prime }\left(x_{j}\right)+\mu a_{j}B_{j+1}^{\prime }\left(x_{j}\right)+b_{j}B_{j+1}\left(x_{j}\right)\right]\gamma_{j+1}=f\left(x_{j}\right),j=0,1,\cdots ,N. \end{array}$$

Eq. (41) is a system of (N+1) linear equations in (N+3) unknowns $\gamma_{-1},\gamma_{0},\gamma_{1},\cdots ,\gamma_{N+1}$. The boundary conditions in (37) become:

(42) $$\gamma_{-1}B_{-1}\left(x_{0}\right)+\gamma_{0}B_{0}\left(x_{0}\right)+\gamma_{1}B_{1}\left(x_{0}\right)=\alpha_{0}.$$

(43) $$\gamma_{N-1}B_{N-1}\left(x_{N}\right)+\gamma_{N}B_{N}\left(x_{N}\right)+\gamma_{N+1}B_{N+1}\left(x_{N}\right)=\alpha_{1}.$$

Eqs. (41), (42) and (43) together represent a system of (N+3) linear equations in (N+3) unknowns. Using Eqs. (29), (31) and (33) in Eq. (42), it is possible to get:

$$\gamma_{-1}\left[\frac{h_{1}}{2(2h_{1}+h_{2})}\right]+\gamma_{0}\left[\frac{h_{1}+h_{2}}{2h_{1}+h_{2}}\right]+\gamma_{1}\left[\frac{h_{1}}{2(2h_{1}+h_{2})}\right]=\alpha_{0}.$$

And this implies that:

(44) $$\gamma_{-1}=\frac{2(2h_{1}+h_{2})}{h_{1}}\alpha_{0}-\frac{2(h_{1}+h_{2})}{h_{1}}\gamma_{0}-\gamma_{1}$$

And using results in (30), (32) and (34) in (43) above it is obtained:

$$\gamma_{N-1}\left[\frac{h_{N}}{2(h_{N-1}+2h_{N})}\right]+\gamma_{N}\left[\frac{h_{N-1}+h_{N}}{h_{N-1}+2h_{N}}\right]+\gamma_{N+1}\left[\frac{h_{N}}{2(h_{N-1}+2h_{N})}\right]=\alpha_{1}.$$

And this implies that:

(45) $$\gamma_{N+1}=\frac{2(h_{N-1}+2h_{N})}{h_{N}}\alpha_{1}-\gamma_{N-1}-\frac{2(h_{N-1}+h_{N})}{h_{N}}\gamma_{N}.$$

For j=0 in Eq. (41), it is obtained:

$$\left[-\varepsilon B_{-1}^{\prime \prime }\left(x_{0}\right)+\mu a_{0}B_{-1}^{\prime }\left(x_{0}\right)+b_{0}B_{-1}\left(x_{0}\right)\right]\gamma_{-1}+\left[-\varepsilon B_{0}^{\prime \prime }\left(x_{0}\right)+\mu a_{0}B_{0}^{\prime }\left(x_{0}\right)+b_{0}B_{0}\left(x_{0}\right)\right]\gamma_{0}+\left[-\varepsilon B_{1}^{\prime \prime }\left(x_{0}\right)+\mu a_{0}B_{1}^{\prime }\left(x_{0}\right)+b_{0}B_{1}\left(x_{0}\right)\right]\gamma_{1}=f\left(x_{0}\right).$$

Then using Eq. (44) in above equation it can be obtained:

$$\begin{array}{cc} & \left[-\varepsilon B_{-1}^{\prime \prime }\left(x_{0}\right)+\mu a_{0}B_{-1}^{\prime }\left(x_{0}\right)+b_{0}B_{-1}\left(x_{0}\right)\right]\left[\frac{2(2h_{1}+h_{2})}{h_{1}}\alpha_{0}-\frac{2(h_{1}+h_{2})}{h_{1}}\gamma_{0}-\gamma_{1}\right]+\left[-\varepsilon B_{0}^{\prime \prime }\left(x_{0}\right)+\mu a_{0}B_{0}^{\prime }\left(x_{0}\right)+b_{0}B_{0}\left(x_{0}\right)\right]\gamma_{0}\\ & \;+\left[-\varepsilon B_{1}^{\prime \prime }\left(x_{0}\right)+\mu a_{0}B_{1}^{\prime }\left(x_{0}\right)+b_{0}B_{1}\left(x_{0}\right)\right]\gamma_{1}=f\left(x_{0}\right). \end{array}$$

Which implies,

(46) $$\begin{array}{cc} & \left\{\left[-\varepsilon B_{0}^{\prime \prime }\left(x_{0}\right)+\mu a_{0}B_{0}^{\prime }\left(x_{0}\right)+b_{0}B_{0}\left(x_{0}\right)\right]-\left[-\varepsilon B_{-1}^{\prime \prime }\left(x_{0}\right)+\mu a_{0}B_{-1}^{\prime }\left(x_{0}\right)+b_{0}B_{-1}\left(x_{0}\right)\right]\frac{2(h_{1}+h_{2})}{h_{1}}\right\}\gamma_{0}\\ & \;+\left\{\left[-\varepsilon B_{1}^{\prime \prime }\left(x_{0}\right)+\mu a_{0}B_{1}^{\prime }\left(x_{0}\right)+b_{0}B_{1}\left(x_{0}\right)\right]-\left[-\varepsilon B_{-1}^{\prime \prime }\left(x_{0}\right)+\mu a_{0}B_{-1}^{\prime }\left(x_{0}\right)+b_{0}B_{-1}\left(x_{0}\right)\right]\right\}\gamma_{1}\\ & =f\left(x_{0}\right)-\left[-\varepsilon B_{-1}^{\prime \prime }\left(x_{0}\right)+\mu a_{0}B_{-1}^{\prime }\left(x_{0}\right)+b_{0}B_{-1}\left(x_{0}\right)\right]\left(\frac{4h_{1}+2h_{2}}{h_{1}}\right)\alpha_{0}. \end{array}$$

For j=1,2,⋯,N−1, it holds:

(47) $$\left[-\varepsilon B_{j-1}^{\prime \prime }\left(x_{j}\right)+\mu a_{j}B_{j-1}^{\prime }\left(x_{j}\right)+b_{j}B_{j-1}\left(x_{j}\right)\right]\gamma_{j-1}+\left[-\varepsilon B_{j}^{\prime \prime }\left(x_{j}\right)+\mu a_{j}B_{j}^{\prime }\left(x_{j}\right)+b_{j}B_{j}\left(x_{j}\right)\right]\gamma_{j}+\left[-\varepsilon B_{j+1}^{\prime \prime }\left(x_{j}\right)+\mu a_{j}B_{j+1}^{\prime }\left(x_{j}\right)+b_{j}B_{j+1}\left(x_{j}\right)\right]\gamma_{j+1}=f\left(x_{j}\right).$$

For j=N in Eq. (41), it is possible to get:

$$\begin{array}{cc} & \left[-\varepsilon B_{N-1}^{\prime \prime }\left(x_{N}\right)+\mu a_{N}B_{N-1}^{\prime }\left(x_{N}\right)+b_{N}B_{N-1}\left(x_{N}\right)\right]\gamma_{N-1}+\left[-\varepsilon B_{N}^{\prime \prime }\left(x_{N}\right)+\mu a_{N}B_{N}^{\prime }\left(x_{N}\right)+b_{N}B_{N}\left(x_{N}\right)\right]\gamma_{N}\\ & \;+\left[-\varepsilon B_{N+1}^{\prime \prime }\left(x_{N}\right)+\mu a_{N}B_{N+1}^{\prime }\left(x_{N}\right)+b_{N}B_{N+1}\left(x_{N}\right)\right]\gamma_{N+1}=f\left(x_{N}\right). \end{array}$$

Using Eq. (45) in the above equation it is possible to get:

$$\begin{array}{cc} & \left[-\varepsilon B_{N-1}^{\prime \prime }\left(x_{N}\right)+\mu a_{N}B_{N-1}^{\prime }\left(x_{N}\right)+b_{N}B_{N-1}\left(x_{N}\right)\right]\gamma_{N-1}+\left[-\varepsilon B_{N}^{\prime \prime }\left(x_{N}\right)+\mu a_{N}B_{N}^{\prime }\left(x_{N}\right)+b_{N}B_{N}\left(x_{N}\right)\right]\gamma_{N}\\ & \;+\left[-\varepsilon B_{N+1}^{\prime \prime }\left(x_{N}\right)+\mu a_{N}B_{N+1}^{\prime }\left(x_{N}\right)+b_{N}B_{N+1}\left(x_{N}\right)\right]\left[\left(\frac{2h_{N-1}+4h_{N}}{h_{N}}\right)\alpha_{1}-\gamma_{N-1}-\left(\frac{2h_{N-1}+2h_{N}}{h_{N}}\right)\gamma_{N}\right]=f\left(x_{N}\right). \end{array}$$

Which implies:

(48) $$\begin{array}{cc} & \left\{\left[-\varepsilon B_{N-1}^{\prime \prime }\left(x_{N}\right)+\mu a_{N}B_{N-1}^{\prime }\left(x_{N}\right)+b_{N}B_{N-1}\left(x_{N}\right)\right]-\left[-\varepsilon B_{N+1}^{\prime \prime }\left(x_{N}\right)+\mu a_{N}B_{N+1}^{\prime }\left(x_{N}\right)+b_{N}B_{N+1}\left(x_{N}\right)\right]\right\}\gamma_{N-1}\\ & \;+\left\{\left[-\varepsilon B_{N}^{\prime \prime }\left(x_{N}\right)+\mu a_{N}B_{N}^{\prime }\left(x_{N}\right)+b_{N}B_{N}\left(x_{N}\right)\right]-\left[-\varepsilon B_{N+1}^{\prime \prime }\left(x_{N}\right)+\mu a_{N}B_{N+1}^{\prime }\left(x_{N}\right)+b_{N}B_{N+1}\left(x_{N}\right)\right]\left(\frac{2h_{N-1}+2h_{N}}{h_{N}}\right)\right\}\gamma_{N}\\ & =f\left(x_{N}\right)-\left[-\varepsilon B_{N+1}^{\prime \prime }\left(x_{N}\right)+\mu a_{N}B_{N+1}^{\prime }\left(x_{N}\right)+b_{N}B_{N+1}\left(x_{N}\right)\right]\left(\frac{2h_{N-1}+4h_{N}}{h_{N}}\right)\alpha_{1}. \end{array}$$

Eqs. (46), (47) and (48) represent a system of (N+1) linear equations in (N+1) unknowns of the form:

Aγ=d

where A is an (N+1) by (N+1) tridiagonal nonsingular matrix, $\gamma ={\left(\gamma_{0},\gamma_{1},\cdots ,\gamma_{N}\right)}^{t}$ is the vector of the unknowns & d is the vector of the right hand side terms. The elements of the tridiagonal matrix $A=(a_{ij})$ are obtained from the coefficients of $\gamma_{0}$ and $\gamma_{1}$ of Eq. (46), from the coefficients of $\gamma_{j-1},\gamma_{j}\;\text{and}\;\gamma_{j+1}$ of Eq. (47) for j=1,2,⋯,N−1 and from the coefficients of $\gamma_{N-1}$ and $\gamma_{N}$ of Eq. (48). The elements of A that are found this way satisfy:

$$|a_{ii}|-\sum_{\underset{j\neq i}{j=1}}^{N-1}|a_{ij}\left|=\right|a_{ii}\left|-\right|a_{i,i-1}\left|-\right|a_{i,i+1}|=b_{i}>0.$$

Hence the coefficient matrix A is strictly diagonally dominant and therefore nonsingular. A is nonsingular in turn implies that the system Aγ=d can be solved for $\gamma_{0},\gamma_{1},\cdots ,\gamma_{N}$ and use Eqs. (44) and (45) to calculate for $\gamma_{-1}$ and $\gamma_{N+1}$. Thus the cubic B-spline collocation method has a unique solution on problem (1)-(2) which is given by Eq. (35).

## Convergence analysis

In this section error bound for cubic B-spline collocation method is found. Let the mesh intervals be denoted by $I_{i}=\left[x_{i-1},x_{i}\right],i=1,2,\cdots ,N$. For p,m∈N,m<p, let:

$$s_{p}^{m}\left(\Omega^{N}\right)=\left\{s\in c^{m}\left[0,1\right]:s{|}_{I_{i}}\in \Pi_{p},\;\text{for}\;i=1,2,\cdots ,N\right\}.$$

and

$$s_{p,0}^{m}\left(\Omega^{N}\right)=\left\{s\in s_{p}^{m}\left(\Omega^{N}\right):s\left(0\right)=s\left(1\right)=0,\right\},$$

where $\Pi_{p}$ is the space of polynomials of degree less than or equal to p.

### $s_{3}^{0}$- interpolation

Let the points $\frac{1}{3}$ and $\frac{2}{3}$ units away from the left end point $x_{i-1}$ of the mesh interval $I_{i}$ denoted respectively by $x_{i-\frac{1}{3}}$ and $x_{i-\frac{2}{3}}$ be defined respectively as:

$$x_{i-\frac{1}{3}}=x_{i-1}+\frac{1}{3}h_{i}=x_{i-1}+\frac{1}{3}\left(x_{i}-x_{i-1}\right)=\frac{2x_{i-1}+x_{i}}{3},$$

and

$$x_{i-\frac{2}{3}}=x_{i-1}+\frac{2}{3}h_{i}=x_{i-1}+\frac{2}{3}\left(x_{i}-x_{i-1}\right)=\frac{x_{i-1}+2x_{i}}{3}.$$

For any function $y\in C^{0}\left[0,1\right]$, the interpolation problem of finding $I_{3}^{0}y\in s_{3}^{0}\left(\Omega^{N}\right)$ is defined by

(49) $${\left(I_{3}^{0}y\right)}_{i}=y_{i},i=0,1,\cdots ,N,{\left(I_{3}^{0}y\right)}_{i-\frac{1}{3}}=y_{i-\frac{1}{3}},{\left(I_{3}^{0}y\right)}_{i-\frac{2}{3}}=y_{i-\frac{2}{3}},$$

where $y_{i},y_{i-\frac{1}{3}}\;$&$\;y_{i-\frac{2}{3}}$ denote the values of the function y at the points $x_{i},x_{i-\frac{1}{3}}\;$&$\;x_{i-\frac{2}{3}}$ of the interval $I_{i}$.

The approach of proof of Theorem (4.1) given in  [26] is used to prove the following theorem.

Theorem 2 *If*$a,b,f\in C^{4}\left[0,1\right]$*, then the interpolation error*$u-I_{3}^{0}u$*for the solution of problem*(1)*-*(2)*defined in*(20)*satisfies:*

(50) $$∥u-I_{3}^{0}u∥\leq CN^{-4}$$

(51) $$\max_{i=1,2,\cdots ,N}\left|{\left(u-I_{3}^{0}u\right)}_{i}^{\prime }\right|\leq C\mu^{-1}N^{-3}$$

(52) $$\max_{i=1,2,\cdots ,N}\left|{\left(u-I_{3}^{0}u\right)}_{i}^{\prime \prime }\right|\leq C\varepsilon^{-1}N^{-2}$$

Lemma 6 *Let* $s\in s_{3}^{0}\left(\Omega^{N}\right)$ *with* $s_{i-\frac{1}{3}}=0,s_{i-\frac{2}{3}}=0,i=1,2,\cdots ,N$ *, then* ${∥s∥}_{I_{i}}\leq 4\max_{i}\left\{\right|s_{i-1}\left|,\right|s_{i}\left|\right\}$ *,* $∥s^{\prime }{∥}_{I_{i}}\leq 2h_{i}\max_{i}\left\{\right|s_{i-1}\left|,\right|s_{i}\left|\right\}$ *and* $∥s^{\prime \prime }{∥}_{I_{i}}\leq \frac{18}{h_{i}}\max_{i}\left\{\right|s_{i-1}\left|,\right|s_{i}\left|\right\}$

Proof On any subinterval $I_{i}=\left[x_{i-1},x_{i}\right]$ of [0,1] take the interpolant $s\in s_{3}^{0}\left(\Omega^{N}\right)$ to be a cubic polynomial interpolating the values $s_{i-1},s_{i-\frac{1}{3}},s_{i-\frac{2}{3}},\;\text{and}\;s_{i}$ respectively in the points $x_{i-1},x_{i-\frac{1}{3}},x_{i-\frac{2}{3}},\;\text{and}\;x_{i}$. The Lagrange form of s is:

$s\left(x\right)=-\frac{9}{h_{i}^{3}}\left(x-x_{i-\frac{1}{3}}\right)\left(x-x_{i-\frac{2}{3}}\right)\left(x-x_{i}\right)s_{i-1}+\frac{9}{h_{i}^{3}}\left(x-x_{i-1}\right)\left(x-x_{i-\frac{1}{3}}\right)\left(x-x_{i-\frac{2}{3}}\right)s_{i},x\in I_{i}.$

This implies $\left|s\left(x\right)|\leq 4\max\{|s_{i-1}|,|s_{i}|\right\}$. And this again implies that

$${∥s∥}_{I_{i}}\leq 4\max\{|s_{i-1}\left|,\right|s_{i}\left|\right\}.$$

Similarly by taking first and second derivatives of s(x), the bounds for $∥s^{\prime }{∥}_{I_{i}}$ and $∥s^{\prime \prime }{∥}_{I_{i}}$ can be found. □

### $s_{3}^{1}$ interpolation.

Given any $y\in C^{1}\left[0,1\right]$, the restriction of y on any subinterval $I_{i}=\left[x_{i-1},x_{i}\right]$ is the unique cubic polynomial $I_{3}^{1}y\in s_{3}^{1}\left(\Omega^{N}\right)$ interpolating the values $y_{i-1},y_{i-\frac{1}{3}},y_{i-\frac{2}{3}},\;\text{and}\;y_{i}$ respectively at $x_{i-1},x_{i-\frac{1}{3}},x_{i-\frac{2}{3}},\;\text{and}\;x_{i}$ and defined by

(53) $${\left(I_{3}^{1}y\right)}_{0}=y_{0},{\left(I_{3}^{1}y\right)}_{i-\frac{1}{3}}=y_{i-\frac{1}{3}},{\left(I_{3}^{1}y\right)}_{i-\frac{2}{3}}=y_{i-\frac{2}{3}},i=1,2,\cdots ,N,\;\text{and}\;{\left(I_{3}^{1}y\right)}_{N}=y_{N}.$$

For any $s\in s_{3}^{1}\left(\Omega^{N}\right)$, considering the data points $\left\{\left(x_{i-1},s_{i-1}\right),\left(x_{i-\frac{1}{3}},s_{i-\frac{1}{3}}\right),\left(x_{i-\frac{2}{3}},s_{i-\frac{2}{3}}\right),\left(x_{i},s_{i}\right)\right\}$ and $\left\{\left(x_{i},s_{i}\right),\left(x_{i+\frac{1}{3}},s_{i+\frac{1}{3}}\right),\left(x_{i+\frac{2}{3}},s_{i+\frac{2}{3}}\right),\left(x_{i+1},s_{i+1}\right)\right\}$ s is found on both $\left[x_{i-1},x_{i}\right]$ and $\left[x_{i},x_{i+1}\right]$ by using Lagrange interpolation and using the fact that $s^{\prime \prime }$ is continuously differentiable at $x_{i}$, it is obtained:

(54) $${\left[Gs\right]}_{i}=-a_{i}s_{i-1}+2s_{i}-c_{i}s_{i+1}=-4a_{i}s_{i-\frac{1}{3}}+5c_{i}s_{i+\frac{1}{3}}+5a_{i}s_{i-\frac{2}{3}}-4c_{i}s_{i+\frac{2}{3}},$$

where $a_{i}=\frac{h_{i+1}^{2}}{h_{i+1}^{2}-h_{i}^{2}}$ and $c_{i}=1-a_{i}$.

Lemma 7 *For all vectors*$s_{i}\in R^{N+1}$*with*$s_{0}=s_{N}=0$*, the operator G is stable, that is,*

$$\max_{i=1,2,\cdots ,N-1}|s_{i}|\leq \max_{i=1,2,\cdots ,N-1}\left|{\left[Gs\right]}_{i}\right|$$

Proof Let $k\in argmax_{i=1,2,\cdots ,N-1}\left|s_{i}\right|$, then ${\left[Gs\right]}_{k}=-a_{k}s_{k-1}+2s_{k}-c_{k}s_{k+1}$.

This implies $2s_{k}={\left[Gs\right]}_{k}+\left(a_{k}+c_{k}\right)s_{k}$, using $s_{k-1},s_{k+1}\approx \frac{s_{k-1}+s_{k+1}}{2}=s_{k}$.

Which gives $2|s_{k}{|\leq |\left[Gs\right]}_{k}|+\left(a_{k}+c_{k}\right)|s_{k}\left|=\right|{\left[Gs\right]}_{k}\left|+\right|s_{k}|,\;\text{since}\;\left(a_{k}+c_{k}\right)=1$. And this implies $|s_{k}{|\leq |\left[Gs\right]}_{k}|.$ Hence it is obtained $\max_{i=1,2,\cdots ,N-1}|s_{i}|\leq \max_{i=1,2,\cdots ,N-1}\left|{\left[Gs\right]}_{i}\right|$ □

Theorem 3 *If*$a,b,f\in C^{4}\left[0,1\right]$*, then the interpolation error*$u-I_{3}^{1}u$*for the solution of problem*(1)*-*(2)*on the graded mesh defined in*(20)*satisfies*

(55) $$\max_{i=0,1,\cdots ,N}\left|{\left(u-I_{3}^{1}u\right)}_{i}\right|\leq C\varepsilon^{-2}N^{-4}$$

(56) $$∥u-I_{3}^{1}u∥\leq C\varepsilon^{-2}N^{-4}$$

(57) $$\max_{i=1,2,\cdots ,N}\left|{\left(u-I_{3}^{1}u\right)}_{i}^{\prime }\right|\leq C\mu^{-1}N^{-3}$$

(58) $$\max_{i=1,2,\cdots ,N}\left|{\left(u-I_{3}^{1}u\right)}_{i}^{\prime \prime }\right|\leq C\varepsilon^{-1}N^{-2}$$

Proof For any $y\in C^{4}\left[0,1\right]$, it holds ${\left(y-I_{3}^{1}y\right)}_{0}=0$ & ${\left(y-I_{3}^{1}y\right)}_{N}=0.$ Using (54) it can be obtained:

$\tau_{y,i}={\left[G\left[y-I_{3}^{1}y\right]\right]}_{i}=-a_{i}{\left[y-I_{3}^{1}y\right]}_{i-1}+2{\left[y-I_{3}^{1}y\right]}_{i}-c_{i}{\left[y-I_{3}^{1}y\right]}_{i+1}$, which implies:

(59) $$\tau_{y,i}={\left[G\left[y-I_{3}^{1}y\right]\right]}_{i}=-a_{i}y_{i-1}+4a_{i}y_{i-\frac{1}{3}}-5a_{i}y_{i-\frac{2}{3}}+2y_{i}-5c_{i}y_{i+\frac{1}{3}}+4c_{i}y_{i+\frac{2}{3}}-c_{i}y_{i+1},i=1,2,\cdots ,N.$$

Using Taylor series expansions of $y_{i-1},y_{i-\frac{1}{3}},y_{i-\frac{2}{3}},y_{i+\frac{1}{3}},y_{i+\frac{2}{3}},\text{and}\;y_{i+1}$ in (59) above it can be obtained:

(60) $$\tau_{y,i}=\frac{49}{3888}h_{i}^{2}h_{i+1}^{2}y_{i}^{\left(iv\right)}$$

After decomposing the interpolation error as $u-I_{3}^{1}u=\left(v-I_{3}^{1}v\right)+\left(w_{L}-I_{3}^{1}w_{L}\right)+\left(w_{R}-I_{3}^{1}w_{R}\right)$ or as $\tau_{u,i}=\tau_{v,i}+\tau_{w_{L},i}+\tau_{w_{R},i}$, the errors in each component are found. To find the error in the regular component for $\left[x_{0},x_{\frac{N}{4}-1}\right]$, it is possible to have:

$$\begin{array}{ccc} |\tau_{v,i}|\leq \frac{49}{3888}h_{i}^{2}h_{i+1}^{2}∥v_{i}^{\left(iv\right)}∥ & \leq & \frac{49}{3888}h_{i}^{2}h_{i+1}^{2}C_{1},\;\text{using}\;\text{inequality}\;\left(16\right)\;\;\text{of}\;\text{Theorem}\;\left(16\right)\\ & \leq & C_{2}h_{i+1}^{4},\text{since}\;h_{i}<h_{i+1}\;\;\text{in}\;\left[x_{0},x_{\frac{N}{4}-1}\right].\\ & \leq & C_{2}v_{0}^{-4}N^{-4}exp\left(\frac{4pv_{0}x_{i+1}}{\tau }\right),\;\text{using}\;\text{inequality}\;\text{in}\;\left(22\right).\\ & \leq & C_{2}N^{-4}exp\left(\frac{4pv_{0}x_{i+1}}{\tau }\right),\;\text{since},\;v_{0}^{-4}<1\;\text{for}\;\mu^{2}\ll \varepsilon .\\ & \leq & CN^{-4} \end{array}$$

For $x_{i}\in \left[x_{\frac{N}{4}},x_{\frac{3N}{4}+1}\right]$ and $x_{i}\in \left[x_{\frac{3N}{4}+2},x_{N}\right]$, $|\tau_{v,i}|\leq CN^{-4}$ is proved in a similar manner. Hence, it is obtained that $|\tau_{v,i}|\leq CN^{-4}$ for $x_{i}\in \left[x_{0},x_{N}\right]$. Using ${\left(v-I_{3}^{1}v\right)}_{i}$ in place of $s_{i}$ in Lemma (7), it can be obtained:

$\max_{i=0,1,\cdots ,N}|{\left(v-I_{3}^{1}v\right)}_{i}|\leq \max_{i=0,1,\cdots ,N}|{\left[G\left(v-I_{3}^{1}v\right)\right]}_{i}|\leq \max_{i=0,1,\cdots ,N}\left|\tau_{v,i}\right|\leq \max_{i=0,1,\cdots ,N}CN^{-4}\leq CN^{-4}.$

That is, $\max_{i=0,1,\cdots ,N}\left|{\left(v-I_{3}^{1}v\right)}_{i}\right|\leq CN^{-4}$.

Following similarly for $w_{L}$ and $w_{R}$ the following bounds can be obtained:

$\max_{i=0,1,\cdots ,N}\left|{\left(w_{L}-I_{3}^{1}w_{L}\right)}_{i}\right|\leq C\varepsilon^{-2}N^{-4}$ and $\max_{i=0,1,\cdots ,N}\left|{\left(w_{R}-I_{3}^{1}w_{R}\right)}_{i}\right|\leq C\varepsilon^{-2}N^{-4}.$

Thus, triangle inequality completes proof for (55).

To prove (56) using triangle inequality it is possible to get

$$\begin{array}{ccc} ∥u-I_{3}^{1}u∥ & \leq & ∥u-I_{3}^{0}u∥+∥I_{3}^{0}u-I_{3}^{1}u∥\leq ∥u-I_{3}^{0}u∥+∥I_{3}^{0}u-I_{3}^{1}{u∥}_{I_{i}},i=1,2,\cdots ,N\\ & \leq & ∥u-I_{3}^{0}u∥+4\max_{i=0,1,\cdots ,N}\left|{\left(I_{3}^{0}u-I_{3}^{1}u\right)}_{i}\right|,\;\text{by}\;\text{Lemma}\;\left(6\right)\\ & \leq & ∥u-I_{3}^{0}u∥+4\max_{i=0,1,\cdots ,N}\left|{\left(u-I_{3}^{1}u\right)}_{i}\right|,\;\text{since}\;{\left(I_{3}^{0}u\right)}_{i}=u_{i}, \end{array}$$

$$\begin{array}{ccc} \Rightarrow ∥u-I_{3}^{1}u∥ & \leq & C_{1}N^{-4}+4C_{2}\varepsilon^{-2}N^{-4},\text{by}\;\;\text{Theorem}\;\left(49\right)\;\text{and}\;\;\text{inequality}\;\left(55\right)\;\text{above},\\ & \leq & C\varepsilon^{-2}N^{-4}.\;\text{And}\;\text{this}\;\text{completes}\;\text{proof}\;\text{for}\;\left(56\right). \end{array}$$

Now to prove (57), using triangle inequality, it is obtained:

$$\begin{array}{cc} \left|{\left(u-I_{3}^{1}u\right)}_{i}^{\prime }\right| & \leq \left|{\left(u-I_{3}^{0}u\right)}_{i}^{\prime }\right|+\left|{\left(I_{3}^{0}u-I_{3}^{1}u\right)}_{i}^{\prime }\right|,\\ & \leq \left|{\left(u-I_{3}^{0}u\right)}_{i}^{\prime }\right|+2h_{i}\max_{i=0,1,\cdots ,N}\left|{\left(I_{3}^{0}u-I_{3}^{1}u\right)}_{i}\right|,\;\text{using}\;\;\text{Lemma}\;\left(49\right),\\ & \leq \left|{\left(u-I_{3}^{0}u\right)}_{i}^{\prime }\right|+2h_{i}\max_{i=0,1,\cdots ,N}\left|{\left(u-I_{3}^{1}u\right)}_{i}\right|,\;\text{since}\;{\left(I_{3}^{0}u\right)}_{i}=u_{i},\\ & \leq C_{1}\mu^{-1}N^{-3}+2h_{i}C_{2}\varepsilon^{-2}N^{-4},\;\;\text{by}\;\text{Theorem}\;\left(49\right)\;\text{and}\;\;\text{inequality}\;\left(55\right),\\ & \leq C\mu^{-1}N^{-3}. \end{array}$$

(61) $$∴\max_{i=1,2,\cdots ,N}\left|{\left(u-I_{3}^{1}u\right)}_{i}^{\prime }\right|\leq C\mu^{-1}N^{-3}$$

To prove (58), using triangle inequality, it is obtained:

$$\begin{array}{ccc} |{\left(u-I_{3}^{1}u\right)}_{i}^{\prime \prime }| & \leq & |{\left(u-I_{3}^{0}u\right)}_{i}^{\prime \prime }\left|+\right|{\left(I_{3}^{0}u-I_{3}^{1}u\right)}_{i}^{\prime \prime }|,\\ & \leq & |{\left(u-I_{3}^{0}u\right)}_{i}^{\prime \prime }|+\frac{18}{h_{i}}\max_{i=0,1,\cdots ,N}\left|{\left(I_{3}^{0}u-I_{3}^{1}u\right)}_{i}\right|,\;\text{using}\;\text{Lemma}\;\left(49\right), \end{array}$$

$$\begin{array}{ccc} \Rightarrow |{\left(u-I_{3}^{1}u\right)}_{i}^{\prime \prime }| & \leq & |{\left(u-I_{3}^{0}u\right)}_{i}^{\prime \prime }|+\frac{18}{h_{i}}\max_{i=0,1,\cdots ,N}\left|{\left(u-I_{3}^{1}u\right)}_{i}\right|,\;\text{since}\;{\left(I_{3}^{0}u\right)}_{i}=u_{i},\\ & \leq & C_{1}\varepsilon^{-1}N^{-2}+\frac{18}{h_{i}}C_{2}\varepsilon^{-2}N^{-4}\;\text{by}\;\text{Theorem}\;\left(49\right)\;\text{and}\;\text{inequality}\;\left(55\right),\\ & \leq & C\varepsilon^{-1}N^{-2}. \end{array}$$

Therefore, $\max_{i=1,2,\cdots ,N}\left|{\left(u-I_{3}^{1}u\right)}_{i}^{\prime \prime }\right|\leq C\varepsilon^{-1}N^{-2}$

### $s_{3}^{2}$ interpolation

For an arbitrary function $y\in C^{2}\left[0,1\right]$, the piecewise cubic interpolation function $I_{3}^{2}y\in s_{3}^{2}\left(\Omega^{N}\right)$ is defined as

(62) $${\left(I_{3}^{2}y\right)}_{0}=y_{0},{\left(I_{3}^{2}y\right)}_{i-\frac{1}{3}}=y_{i-\frac{1}{3}},{\left(I_{3}^{2}y\right)}_{i-\frac{2}{3}}=y_{i-\frac{2}{3}},{\left(I_{3}^{2}y\right)}_{N}=y_{N}.$$

For any function $s\in s_{3}^{2}\left(\Omega^{N}\right)$ it can be obtained:

(63) $${\left[Ms\right]}_{i}=-a_{i}s_{i-1}+2s_{i}-c_{i}s_{i+1}=-4a_{i}s_{i-\frac{1}{3}}+5c_{i}s_{i+\frac{1}{3}}+5a_{i}s_{i-\frac{2}{3}}-4c_{i}s_{i+\frac{2}{3}}.$$

Derivation of (63) is similar to derivation of (54).

Theorem 4 *If*$a,b,f\in C^{4}\left[0,1\right]$*, then the interpolation error*$u-I_{3}^{2}u$*for the solution of problem*(1)*-*(2)*on the graded mesh defined in*(20)*satisfies*

(64) $$\max_{i=0,1,\cdots ,N}\left|{\left(u-I_{3}^{2}u\right)}_{i}\right|\leq C\varepsilon^{-2}N^{-4}$$

(65) $$∥u-I_{3}^{2}u∥\leq C\varepsilon^{-2}N^{-4}$$

(66) $$\max_{i=1,2,\cdots ,N}\left|{\left(u-I_{3}^{2}u\right)}_{i}^{\prime }\right|\leq C\mu^{-1}N^{-3}$$

(67) $$\max_{i=1,2,\cdots ,N}\left|{\left(u-I_{3}^{2}u\right)}_{i}^{\prime \prime }\right|\leq C\varepsilon^{-1}N^{-2}$$

Proof is similar to proof in Theorem (3).

### The error bound

Let $\tilde{s}$ given by

$\tilde{s}\left(x\right)=\sum_{i=-1}^{N+1}\omega_{i}B_{i}\left(x\right)$, be the approximate solution of problem (1)-(2) such that

(68) $${\left[L\tilde{s}\right]}_{i}=f_{i},i=0,1,\cdots ,N,\;\text{and}$$

(69) $${\tilde{s}}_{0}=\alpha_{0},{\tilde{s}}_{N}=\alpha_{1},$$

where $\omega_{i}$’s are unknown real coefficients to be determined by using boundary conditions and collocation method. One can show that (68) is equivalent to

(70) $${\left[L\omega \right]}_{i}=f_{i},i=0,1,\cdots ,N,$$

where $\omega ={\left(\omega_{-1},\omega_{0},\cdots ,\omega_{N+1}\right)}^{T}\in R^{N+3}$ and

(71) $$\begin{array}{cc} & {\left[L\omega \right]}_{i}=-\varepsilon \left[\frac{6(\omega_{i+1}-\omega_{i})}{\left(h_{i+2}+h_{i+1}+h_{i}\right)\left(h_{i+1}+h_{i}\right)}-\frac{6(\omega_{i}-\omega_{i-1})}{\left(h_{i+1}+h_{i}+h_{i-1}\right)\left(h_{i+1}+h_{i}\right)}\right]+\mu a_{i}\left[\frac{3h_{i}\left(\omega_{i+1}-\omega_{i}\right)}{\left(h_{i+2}+h_{i+1}+h_{i}\right)\left(h_{i+1}+h_{i}\right)}+\frac{3h_{i+1}\left(\omega_{i}-\omega_{i-1}\right)}{\left(h_{i+1}+h_{i}+h_{i-1}\right)\left(h_{i+1}+h_{i}\right)}\right]\\ & \;+b_{i}\left[\frac{h_{i}^{2}\left(2\omega_{i+1}-\omega_{i}\right)}{2\left(h_{i+2}+h_{i+1}+h_{i}\right)\left(h_{i+1}+h_{i}\right)}+\left(1-\frac{h_{i}^{2}}{2\left(h_{i+2}+h_{i+1}+h_{i}\right)\left(h_{i+1}+h_{i}\right)}-\frac{h_{i+1}^{2}}{2\left(h_{i+1}+h_{i}+h_{i-1}\right)\left(h_{i+1}+h_{i}\right)}\right)\omega_{i}+\frac{h_{i+1}^{2}\left(2\omega_{i-1}-\omega_{i}\right)}{2\left(h_{i+2}+h_{i+1}+h_{i}\right)\left(h_{i+1}+h_{i}\right)}\right] \end{array}$$

Set $h_{0}=h_{N+1}=h_{N+2}=0$. The first and second equations in (69) are respectively equivalent to

(72) $$\omega_{-1}=\frac{2(2h_{1}+h_{2})}{h_{1}}\alpha_{1}-\frac{2\omega_{0}\left(h_{1}+h_{2}\right)}{h_{1}}-\omega_{1}$$

and

(73) $$\omega_{N+1}=\frac{\left(h_{N+1}+h_{N}\right)\left(h_{N+2}+h_{N+1}+h_{N}\right)}{h_{N}^{2}}\alpha_{1}-\frac{\left(h_{N+2}+h_{N+1}+h_{N}\right)h_{N+1}^{2}}{h_{N}^{2}\left(h_{N+1}+h_{N}+h_{N-1}\right)}\omega_{N-1}-\left[\frac{h_{N+2}+h_{N}}{h_{N}^{2}}+\frac{\left(h_{N+2}+h_{N+1}+h_{N}\right)\left(h_{N}+h_{N-1}\right)}{h_{N}^{2}\left(h_{N+1}+h_{N}+h_{N-1}\right)}\right]\omega_{N}.$$

Theorem 5 *The operator*L*is stable with*$∥\gamma ∥\leq \frac{1}{4\beta }∥L\gamma ∥,\;\text{for}\;\text{all}\;\gamma \in R_{0}^{N+3},$*where*$R_{0}^{N+3}:=\left\{\gamma \in R^{N+3}:\gamma_{0}=\gamma_{N+1}=0\right\}$*.*

Proof (74) $$\text{Let}\;\;m_{i}=b_{i}\left(1-\frac{h_{i}^{2}}{2\left(h_{i+2}+h_{i+1}+h_{i}\right)\left(h_{i+1}+h_{i}\right)}-\frac{h_{i+1}^{2}}{2\left(h_{i+1}+h_{i}+h_{i-1}\right)\left(h_{i+1}+h_{i}\right)}\right),i=0,1,\cdots ,N.$$

Clearly it is possible to have:

(75) $$\frac{h_{i}^{2}}{2\left(h_{i+2}+h_{i+1}+h_{i}\right)\left(h_{i+1}+h_{i}\right)}<\frac{1}{2},\;\frac{h_{i+1}^{2}}{2\left(h_{i+1}+h_{i}+h_{i-1}\right)\left(h_{i+1}+h_{i}\right)}<\frac{1}{2},$$

This implies $m_{i}>0,\forall i=0,1,\cdots ,N$ and $m_{i}=b_{i}\left(1-\frac{h_{i}^{2}}{2\left(h_{i+2}+h_{i+1}+h_{i}\right)\left(h_{i+1}+h_{i}\right)}-\frac{h_{i+1}^{2}}{2\left(h_{i+1}+h_{i}+h_{i-1}\right)\left(h_{i+1}+h_{i}\right)}\right)>b_{i}$

Which gives

(76) $$m_{i}>b_{i},\frac{1}{m_{i}}<\frac{1}{b_{i}}.$$

For any vector $\gamma \in R_{0}^{N+3}$ and for each i=0,1,⋯,N, let the operator Λ be defined by

$${\left[\Lambda \gamma \right]}_{i}=-\frac{\varepsilon }{m_{i}}\left[\frac{6(\gamma_{i+1}-\gamma_{i})}{\left(h_{i+2}+h_{i+1}+h_{i}\right)\left(h_{i+1}+h_{i}\right)}-\frac{6(\gamma_{i}-\gamma_{i-1})}{\left(h_{i+1}+h_{i}+h_{i-1}\right)\left(h_{i+1}+h_{i}\right)}\right]+\frac{\mu a_{i}}{m_{i}}\left[\frac{3h_{i}\left(\gamma_{i+1}-\gamma_{i}\right)}{\left(h_{i+2}+h_{i+1}+h_{i}\right)\left(h_{i+1}+h_{i}\right)}+\frac{3h_{i+1}\left(\gamma_{i}-\gamma_{i-1}\right)}{\left(h_{i+1}+h_{i}+h_{i-1}\right)\left(h_{i+1}+h_{i}\right)}\right]+\gamma_{i}.$$

Since $m_{i}>0,\forall i=0,1,\cdots ,N$, Λ is well defined. After using $\gamma_{0}=\gamma_{N+1}=0$, Λ becomes a square matrix. Moreover the fact that

$$\begin{array}{cc} {\left[\Lambda \gamma \right]}_{i} & =\left[\frac{-6\varepsilon }{m_{i}\left(h_{i+1}+h_{i}+h_{i-1}\right)\left(h_{i+1}+h_{i}\right)}-\frac{3\mu h_{i+1}a_{i}}{m_{i}\left(h_{i+1}+h_{i}+h_{i-1}\right)\left(h_{i+1}+h_{i}\right)}\right]\gamma_{i-1}+[\frac{6\varepsilon }{m_{i}\left(h_{i+2}+h_{i+1}+h_{i}\right)\left(h_{i+1}+h_{i}\right)}\\ & \;+\frac{6\varepsilon }{m_{i}\left(h_{i+1}+h_{i}+h_{i-1}\right)\left(h_{i+1}+h_{i}\right)}-\frac{3\mu h_{i}a_{i}}{m_{i}\left(h_{i+2}+h_{i+1}+h_{i}\right)\left(h_{i+1}+h_{i}\right)}+\frac{3\mu h_{i+1}a_{i}}{m_{i}\left(h_{i+1}+h_{i}+h_{i-1}\right)\left(h_{i+1}+h_{i}\right)}]\gamma_{i}\\ & \;+\left[\frac{-6\varepsilon }{m_{i}\left(h_{i+2}+h_{i+1}+h_{i}\right)\left(h_{i+1}+h_{i}\right)}+\frac{3\mu h_{i}a_{i}}{m_{i}\left(h_{i+2}+h_{i+1}+h_{i}\right)\left(h_{i+1}+h_{i}\right)}\right]\gamma_{i+1}, \end{array}$$

implies that the non-diagonal entries of Λ are all non-positive if $3\mu h_{i}a_{i}\leq 6\varepsilon$ for each i and the row sums of Λ are at least 1. Thus, by M-criterion in [14] it can be obtained $∥\Lambda^{-1}∥<1.$ In addition note that:

${\left[\Lambda \gamma \right]}_{i}=\frac{{\left[L\gamma \right]}_{i}}{m_{i}}-\frac{b_{i}}{m_{i}}\left[\frac{h_{i}^{2}\left(2\gamma_{i+1}-\gamma_{i}\right)}{2\left(h_{i+2}+h_{i+1}+h_{i}\right)\left(h_{i+1}+h_{i}\right)}+\frac{h_{i+1}^{2}\left(2\gamma_{i-1}-\gamma_{i}\right)}{2\left(h_{i+1}+h_{i}+h_{i-1}\right)\left(h_{i+1}+h_{i}\right)}\right],i=0,1,\cdots ,N$ and $\gamma =\Lambda^{-1}\left(\Lambda \gamma \right)$. The latter implies that: $∥\gamma ∥=∥\Lambda^{-1}\left(\Lambda \gamma \right)∥\leq ∥\Lambda^{-1}∥\cdot ∥\Lambda \gamma ∥\leq ∥\Lambda \gamma ∥$, since $∥\Lambda^{-1}∥\leq 1$.

Which implies

(77) $$∥\gamma ∥\leq \max_{i=0,1,\cdots ,N}\left|\frac{{\left[L\gamma \right]}_{i}}{m_{i}}-\frac{b_{i}}{m_{i}}\left[\frac{h_{i}^{2}\left(2\gamma_{i+1}-\gamma_{i}\right)}{2\left(h_{i+2}+h_{i+1}+h_{i}\right)\left(h_{i+1}+h_{i}\right)}+\frac{h_{i+1}^{2}\left(2\gamma_{i-1}-\gamma_{i}\right)}{2\left(h_{i+1}+h_{i}+h_{i-1}\right)\left(h_{i+1}+h_{i}\right)}\right]\right|$$

But using (75) and (76) it holds:

$$\left|\frac{{\left[L\gamma \right]}_{i}}{m_{i}}-\frac{b_{i}}{m_{i}}\left[\frac{h_{i}^{2}\left(2\gamma_{i+1}-\gamma_{i}\right)}{2\left(h_{i+2}+h_{i+1}+h_{i}\right)\left(h_{i+1}+h_{i}\right)}+\frac{h_{i+1}^{2}\left(2\gamma_{i-1}-\gamma_{i}\right)}{2\left(h_{i+1}+h_{i}+h_{i-1}\right)\left(h_{i+1}+h_{i}\right)}\right]\right|\leq \left|\frac{{\left[L\gamma \right]}_{i}}{b_{i}}\right|-3∥\gamma ∥.$$

Finally using this result in (77) it is obtained

$$∥\gamma ∥\leq \frac{1}{4\beta }∥L\gamma ∥$$

Theorem 6 *Let*u(x)*and*$\tilde{s}\left(x\right)$*be the exact and cubic B-spline collocation approximate solutions respectively for the problem*(1)*-*(2)*, then the parameters uniform error estimate is*$∥u-\tilde{s}∥\leq CN^{-2}$*.*

Proof To bound the error $∥u-\tilde{s}∥$, let $I_{3}^{2}u$ be the B-spline interpolant to the solution u. Let $\tilde{s}\left(x\right)=\sum_{i=-1}^{N+1}\omega_{i}B_{i}\left(x\right)$ and $I_{3}^{2}u=\sum_{i=-1}^{N+1}{\tilde{\omega }}_{i}B_{i}\left(x\right)$. Then it is clearly true that: $\left(\omega -\tilde{\omega }\right)\in R_{0}^{N+3}$ and ${\left[L\left(\omega -\tilde{\omega }\right)\right]}_{i}=L{\left(\tilde{s}-I_{3}^{2}u\right)}_{i}=\varepsilon {\left(I_{3}^{2}u-u\right)}_{i}^{\prime \prime }-\mu a_{i}{\left(I_{3}^{2}u-u\right)}_{i}^{\prime }$. Now using Theorem (5) for $\gamma =(\omega -\tilde{\omega })$ it is obtained:

$$\begin{array}{ccc} ∥\omega -\tilde{\omega }∥ & \leq & \frac{1}{4\beta }∥L\left(\omega -\tilde{\omega }\right)∥=\frac{1}{4\beta }\max_{i=1,2,\cdots ,N}|{\left[L\left(\omega -\tilde{\omega }\right)\right]}_{i}|\leq \frac{1}{4\beta }\max_{i=1,2,\cdots ,N}\left|\varepsilon {\left(I_{3}^{2}u-u\right)}_{i}^{\prime \prime }-\mu a_{i}{\left(I_{3}^{2}u-u\right)}_{i}^{\prime }\right|\\ & \leq & \frac{1}{4\beta }\max_{i=1,2,\cdots ,N}\{|\varepsilon {\left(I_{3}^{2}u-u\right)}_{i}^{\prime \prime }|+|\mu a_{i}{\left(I_{3}^{2}u-u\right)}_{i}^{\prime }\left|\right\}\\ & \leq & \frac{\varepsilon }{4\beta }\max_{i=1,2,\cdots ,N}|{\left(I_{3}^{2}u-u\right)}_{i}^{\prime \prime }|+\frac{\mu a_{i}}{4\beta }\max_{i=1,2,\cdots ,N}\left|{\left(I_{3}^{2}u-u\right)}_{i}^{\prime }\right|\\ & \leq & \frac{\varepsilon }{4\beta }\left(C_{1}\varepsilon^{-1}\right)N^{-2}+\frac{\mu a_{i}}{4\beta }\left(C_{2}\mu^{-1}N^{-3}\right),\;\;\text{by}\;\text{inequalities}\;\left(66\right)\;\;\text{and}\;\left(67\right)\;\;\text{of}\;\text{Theorem}\;\left(4\right).\\ & \leq & CN^{-2}. \end{array}$$

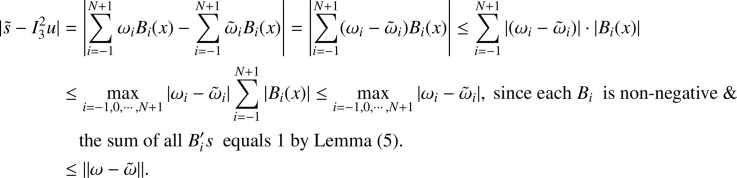

Thus, it holds $∥I_{3}^{2}u-\tilde{s}∥\leq ∥\omega -\tilde{\omega }∥\leq CN^{-2}$ and using triangle inequality it is possible to get:

$$\begin{array}{ccc} ∥u-\tilde{s}∥ & \leq & ∥u-I_{3}^{2}u∥+∥I_{3}^{2}u-\tilde{s}∥,\\ & \leq & C_{1}\varepsilon^{-2}N^{-4}+C_{2}N^{-2},\;\text{Using}\;\text{theorem}\;\left(50\right)\;\text{and}\;\text{above}\;\text{inequality}\\ & \leq & C_{1}N^{-2}+C_{2}N^{-2},\;\text{assuming}\;\varepsilon \leq N^{-1}\\ & \leq & CN^{-2}. \end{array}$$

Hence, $∥u-\tilde{s}∥\leq CN^{-2}$ shows that $∥u-\tilde{s}∥\to 0$ as N→∞, and this depicts that the method is theoretically parameters uniform convergent of order two. □

## Numerical examples

In this section the proposed numerical scheme is applied on a few test problems with two small parameters having two boundary layers to validate the theoretical results.

Example 1 Let $-\epsilon u^{\prime \prime }\left(x\right)+\mu u^{\prime }\left(x\right)+u\left(x\right)=\cos\left(\pi x\right),\;x\in \left(0,1\right),\;u\left(0\right)=u\left(1\right)=0,$

The exact solution of this equation is $u\left(x\right)=a_{1}\cos\left(\pi x\right)+a_{2}\sin\left(\pi x\right)+a_{3}e^{\lambda_{1}x}+a_{4}e^{-\lambda_{2}\left(1-x\right)}$, where $a_{1}=\frac{\epsilon \pi^{2}+1}{\mu^{2}\pi^{2}+{\left(\epsilon \pi^{2}+1\right)}^{2}},\;a_{2}=\frac{\mu \pi }{\mu^{2}\pi^{2}+{\left(\epsilon \pi^{2}+1\right)}^{2}},\;a_{3}=-a_{1}\frac{1+e^{-\lambda_{2}}}{1-e^{\lambda_{1}-\lambda_{2}}},$$a_{4}=a_{1}\frac{1+e^{\lambda_{1}}}{1-e^{\lambda_{1}-\lambda_{2}}},\;\lambda_{1,2}=\frac{\mu \mp \sqrt{\mu^{2}+4\epsilon }}{2\epsilon }$.

Example 2 Consider the second test problem with constant coefficients $-\epsilon u^{\prime \prime }\left(x\right)-\mu u^{\prime }\left(x\right)+u\left(x\right)=e^{1-x},\;x\in \left(0,1\right),\;u\left(0\right)=u\left(1\right)=0$.

The exact solution for this equation is $u\left(x\right)=\frac{1}{\epsilon -\mu -1}\left[\frac{e-e^{a_{1}}}{1-e^{\frac{-\sqrt{\mu^{2}+4\epsilon }}{\epsilon }}}e^{-a_{2}x}-e^{1-x}+\frac{e^{1-a_{2}}-1}{e^{\frac{-\sqrt{\mu^{2}+4\epsilon }}{\epsilon }}-1}e^{a_{1}\left(1-x\right)}\right],$ where $a_{1}=\frac{\mu -\sqrt{\mu^{2}+4\epsilon }}{2\epsilon },a_{2}=\frac{\mu +\sqrt{\mu^{2}+4\epsilon }}{2\epsilon }.$

Example 3 Now consider $-\epsilon u^{\prime \prime }\left(x\right)+\mu \left(3-2x^{2}\right)u^{\prime }\left(x\right)+u\left(x\right)={\left(1+x\right)}^{2},\;x\in \left(0,1\right),\;u\left(0\right)=u\left(1\right)=0,$ which is variable coefficient problem.

For Examples (1) and (2) for any value of N the maximum pointwise absolute errors $E_{\epsilon ,\mu }^{N}$ and the order of convergence $R_{\epsilon ,\mu }^{N}$ are calculated by:

(78) $$E_{\epsilon ,\mu }^{N}=∥u_{\epsilon ,\mu }-U_{\epsilon ,\mu }{∥}_{\Omega^{N}}=\max_{1\leq i\leq N-1}\left|u_{i}-U_{i}^{N}\right|,R_{\epsilon ,\mu }^{N}=\log_{2}\left(\frac{E_{\epsilon ,\mu }^{N}}{E_{\epsilon ,\mu }^{2N}}\right),$$

where $u_{\epsilon ,\mu }$ and $U_{\epsilon ,\mu }^{N}$ are the exact solution and the numerical solution of the problems in the examples. The exact solution for Example (3) is not available. As the exact solution is not available for Example (3), the accuracy of its numerical solution will be computed using the double mesh principle. This principle is defined as follows: For any fixed value of N, the maximum point-wise error $E_{\epsilon ,\mu }^{N}$ of the numerical solution $U(x_{i})$ will be calculated by

(79) $$E_{\epsilon ,\mu }^{N}=∥U_{i}^{N}-U_{2i}^{2N}∥=\max_{1\leq i\leq N-1}\left|U_{i}^{N}-U_{2i}^{2N}\right|,$$

where $U_{i}^{N}$ is the numerical solution at the node $x_{i}$ of the original graded mesh with N number of mesh intervals and $U_{2i}^{2N}$ is the numerical solution at the node $x_{2i}$ on a mesh containing 2N number of mesh intervals (i.e. a mesh containing the mesh points of the original graded mesh and its mid points given by $x_{j+\frac{1}{2}}=\left(x_{j+1}+x_{j}\right)/2,\;j=0,1,\cdots ,N-1$).

And the order of convergence $R_{\epsilon ,\mu }^{N}$ is calculated by

(80) $$R_{\epsilon ,\mu }^{N}=\log_{2}\left(\frac{E_{\epsilon ,\mu }^{N}}{E_{\epsilon ,\mu }^{2N}}\right).$$

## Results & discussion

**Fig. 1:**
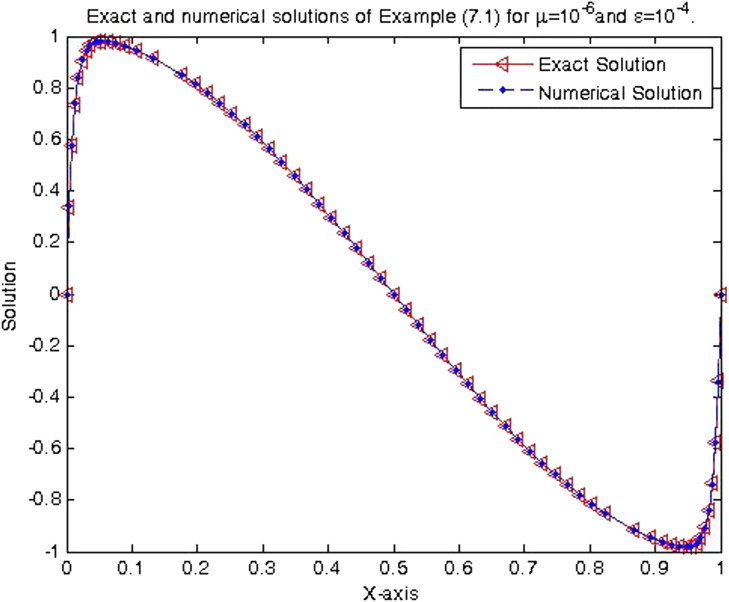
Exact & numerical solutions of Example (1) for $\mu =10^{-6}$ & $\varepsilon =10^{-4}$.

**Fig. 2:**
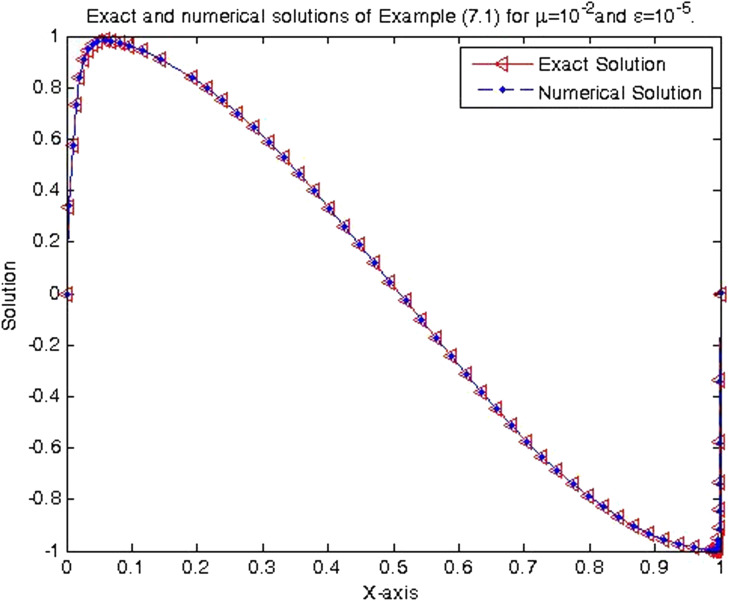
Exact and numerical solutions of Example (1) for $\mu =10^{-2}$& $\varepsilon =10^{-5}$.

**Fig. 3:**
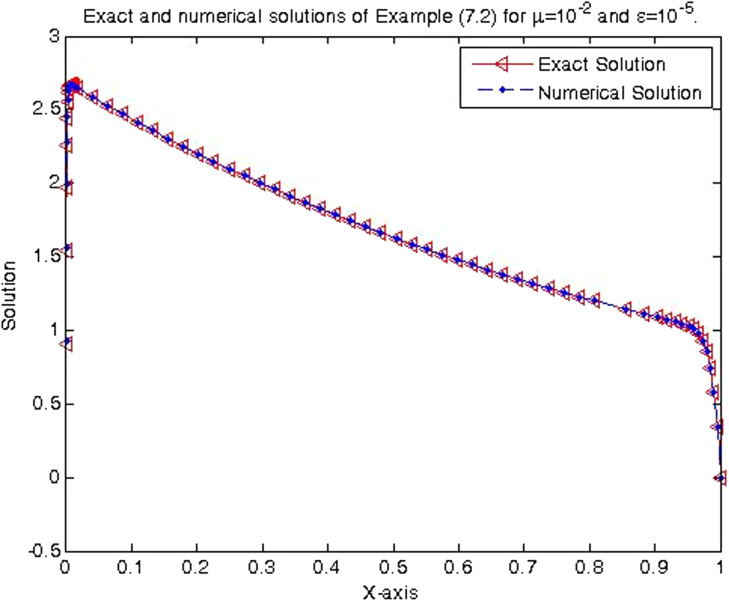
Exact and numerical solutions of Example (2) for $\mu =10^{-2}$& $\varepsilon =10^{-5}$.

**Fig. 4:**
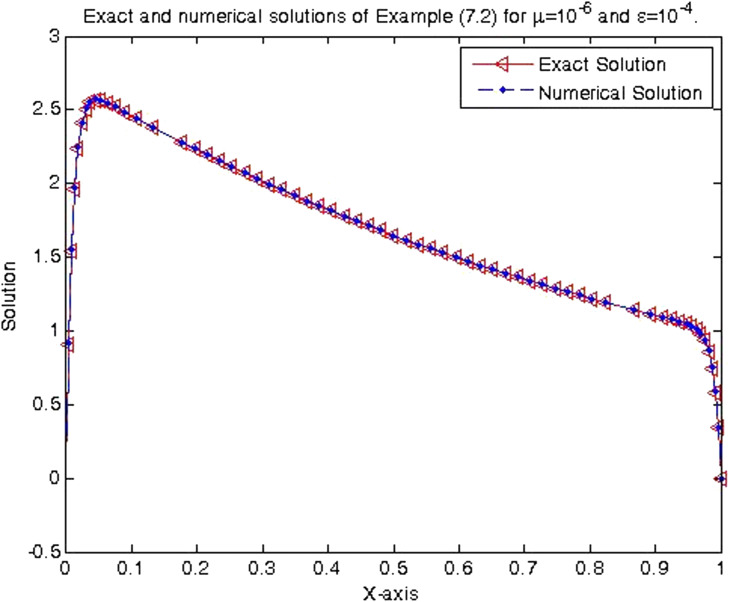
Exact and numerical solutions of Example (2) for $\mu =10^{-6}$& $\varepsilon =10^{-4}$.

**Fig. 5:**
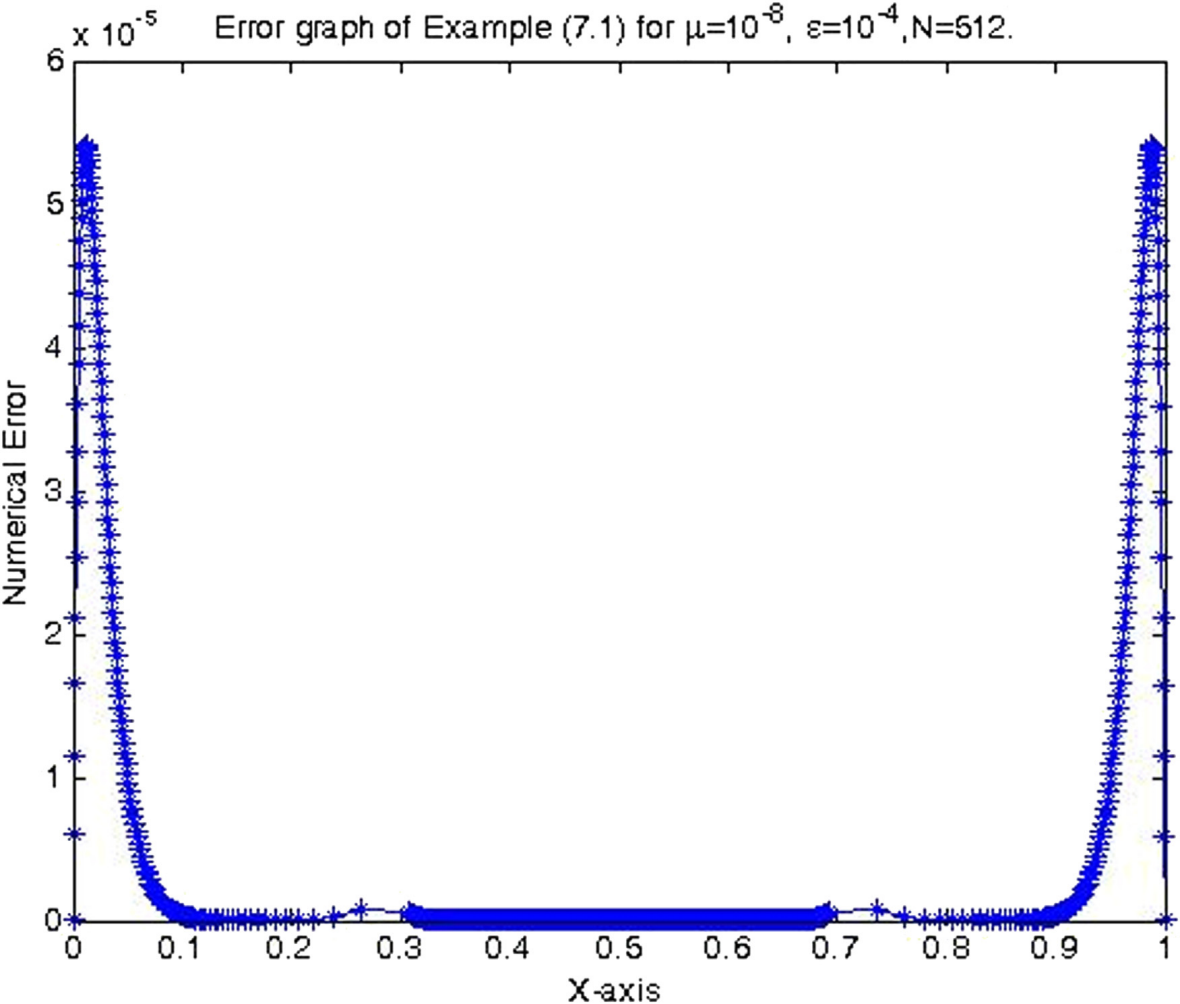
Error graph of Example (1) when $\mu =10^{-8}$, $\varepsilon =10^{-4}$ & N=512.

**Fig. 6:**
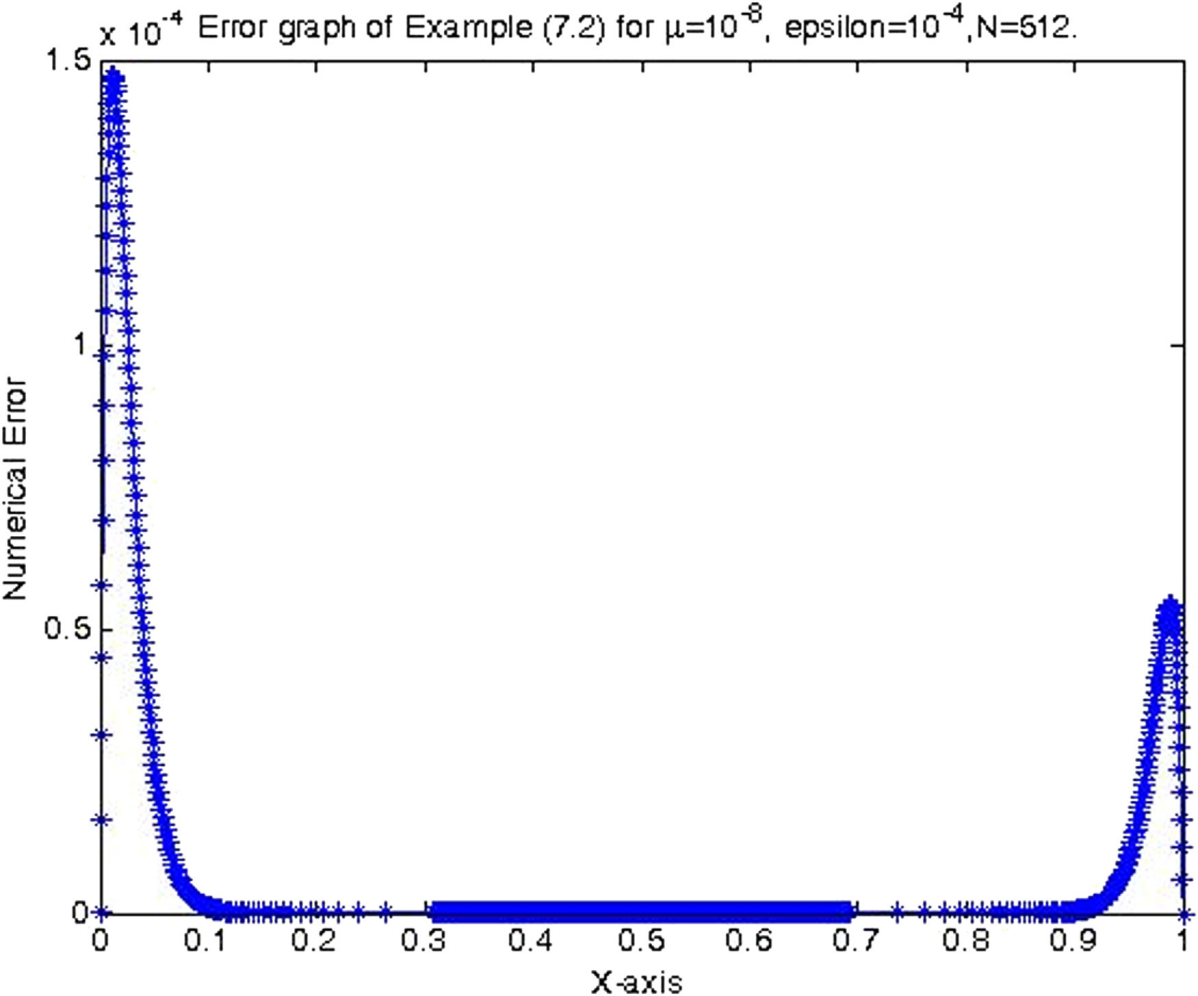
Error graph of Example (2) when $\mu =10^{-8}$, $\varepsilon =10^{-4}$ & N=512.

**Fig. 7:**
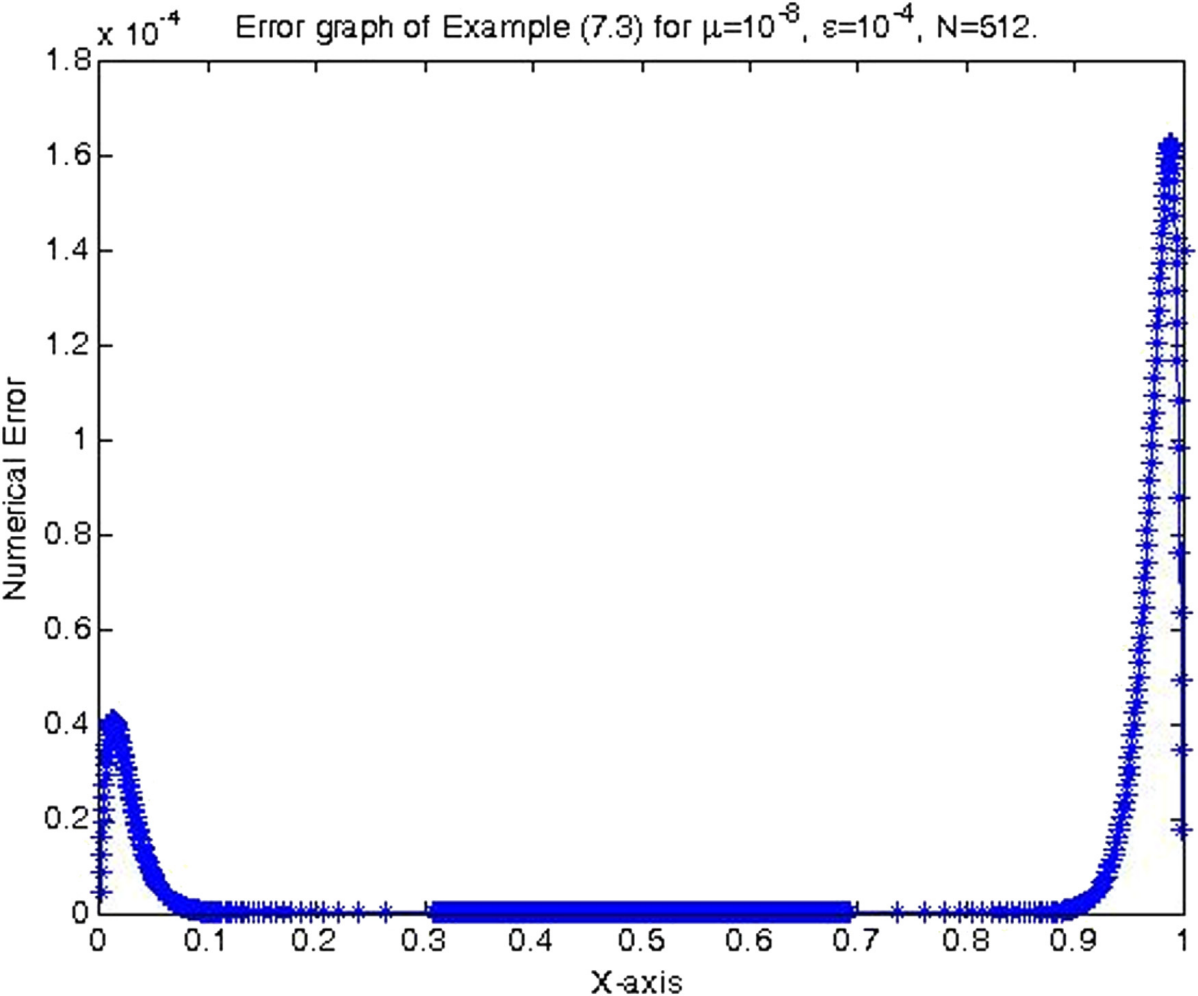
Error graph of Example (3) when $\mu =10^{-8}$, $\varepsilon =10^{-4}$ & N=512.

**Fig. 8:**
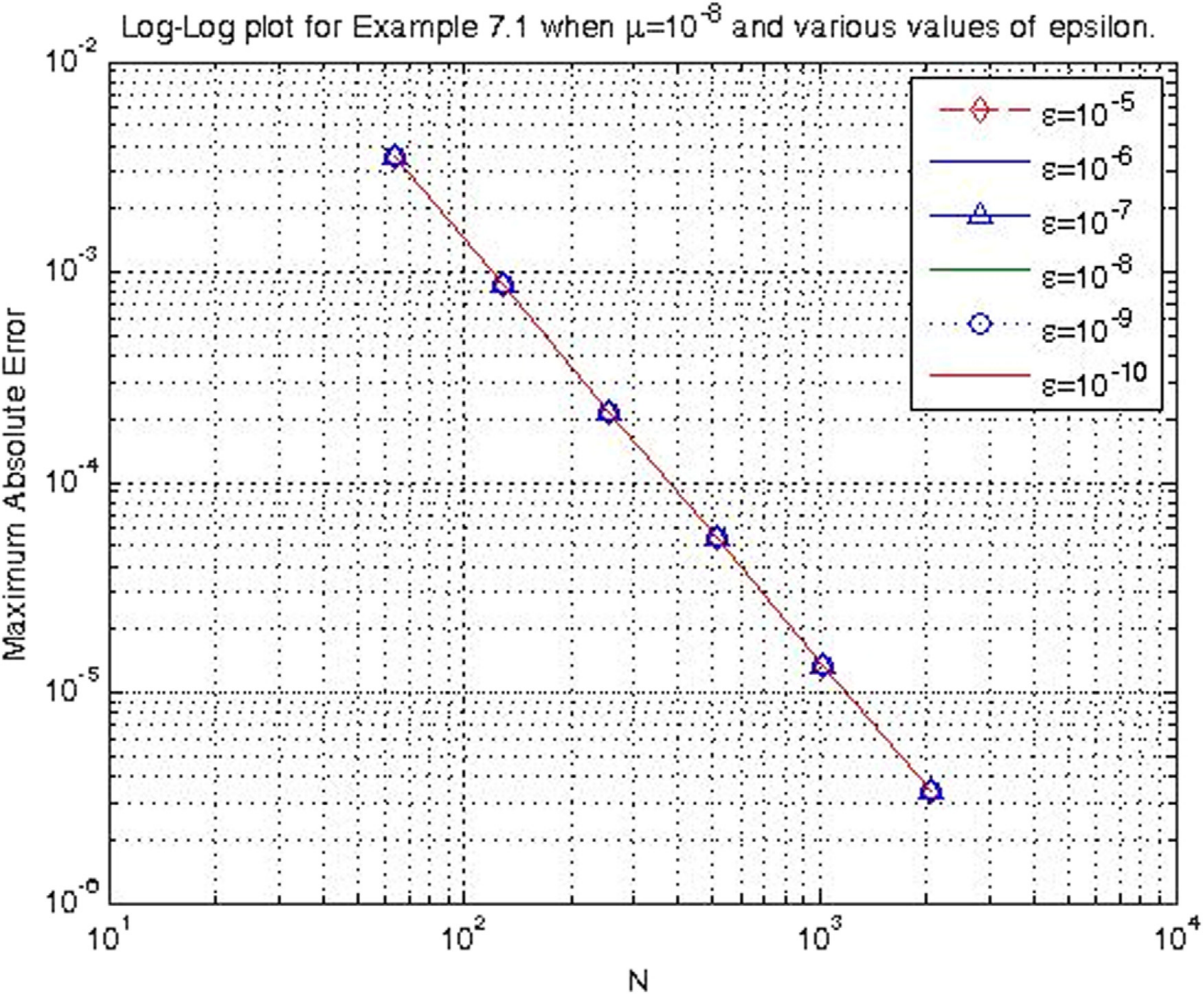
Log-Log plot of Example (1) when $\mu =10^{-8}$ & various values of ε.

In the previous section, the proposed cubic B-spline collocation method on graded mesh is applied on three test problems known in the literature to demonstrate its efficiency in obtaining the numerical solution of a class of TPSPBVPs of the form (1)-(2). The maximum point-wise errors and the orders of convergence for examples Example 1, Example 2 and (3) for fixed values of μ, different values of ϵ and for N={64,128,256,512,1024,2048} are shown by Table 1, Table 2, Table 3, Table 4, Table 5–6. It is observed from these results that as N increases the maximum absolute errors decrease independently of the values of the perturbation parameters, which shows the parameters uniform convergence of the method. The log-log plot of the maximum pointwise absolute errors vs N for Example (1)is given in Fig. 8. In this plot the lines corresponding to the various values of ε are overlapping and it seems that the plot has only a single line. As one moves from left to right (as N increases) along this plot the lines are getting down (pointwise maximum absolute errors are decreasing) independently of the values of the perturbation parameters. Further, from the plot it can be seen that the rate of convergence is approximately two (this can also be seen from Tables 1–6. Hence the log-log plot also illustrates the parameters uniform convergence of the present method and that its rate of convergence is two. Hence Tables 1–6 and Fig. 8 validate the theoretical finding that the present method is parameters uniform convergent of order two. It can be observed that the graphs of numerical and exact solutions are very close to each other from Fig. 1, Fig. 2, Fig. 3–4. Further from Fig. 5, Fig. 6–7, it can be observed that the numerical errors in the approximate solutions are very small. For example as it can be seen from Fig. 5, the maximum numerical error, when $\mu =10^{-8},\varepsilon =10^{-4}$ and N=512 for Example (1) is about $5.5\times 10^{-5}$ which is a small number. Hence Figs. 1–7 depict that the present method approximates the solution very well. The proposed method is also compared with three other methods existing in the literature. Table 7 shows comparison of the present method with method in [22] for Example (1) when $\mu =10^{-8}$ and N=128. When the maximum pointwise absolute errors and the rate of convergence for each various values of ε are compared, it is seen that the errors in the present method are smaller than those in method [22] and the rate of convergence in the present method are higher than those in method [22]. For example for $\varepsilon =10^{-6}$, the error in the present method which is $8.7025e^{-04}$ is smaller than the error $9.0080e^{-03}$ in method [22], and the rate of convergence 2.0037 in the present method is higher than the rate of convergence 1.6351 in [22], and so on. Smaller error shows that the method is more accurate and a higher rate of convergence shows that the method is quicker in converging to the exact solution. Hence Table 7 shows that the proposed method is more accurate and having a higher rate of convergence than the method in [22], that is, the cubic B-spline collocation method on graded mesh (present method) is better (more efficient) than the cubic B-spline collocation method on Shishkin mesh (method in [22]). Similarly, Table 8 shows that the present method is more accurate and having a higher rate of convergence (more efficient) than the method in [31] already existing in the literature. Although it is possible to observe in a similar way that the method in  [26] is better in accuracy than the present method from Table 9, it can also be seen from the same table that the present method has higher rate of convergence than the present method. That is, the present method has improved both accuracy and rate of convergence of the methods in [22] and in [31] whereas it improved only the rate of convergence (not accuracy) of the method in [26].

**Table 1:** $E_{\varepsilon ,\mu }^{N}$ and $R_{\varepsilon ,\mu }^{N}$ Example (1) when $\mu =10^{-4}$ and various values of ε .

ε↓	N					
	64	128	256	512	1024	2048
$10^{-7}$	$\begin{array}{c} 7.6152e-03\\ 2.0092 \end{array}$	$\begin{array}{c} 1.8917e-03\\ 1.9967 \end{array}$	$\begin{array}{c} 4.7402e-04\\ 2.0007 \end{array}$	$\begin{array}{c} 1.1845e-04\\ 2.0004 \end{array}$	$\begin{array}{c} 2.9605e-05\\ 2.0073 \end{array}$	$\begin{array}{c} 7.3639e-06 \end{array}$
$10^{-8}$	$\begin{array}{c} 9.1578e-03\\ 2.1256 \end{array}$	$\begin{array}{c} 2.0985e-03\\ 2.0006 \end{array}$	$\begin{array}{c} 5.2442e-04\\ 2.0006 \end{array}$	$\begin{array}{c} 1.31055e-04\\ 1.9985 \end{array}$	$\begin{array}{c} 3.2795e-05\\ 1.9457 \end{array}$	$\begin{array}{c} 8.5130e-06 \end{array}$
$10^{-9}$	$\begin{array}{c} 7.6152e-03\\ 1.3709 \end{array}$	$\begin{array}{c} 2.9443e-03\\ 2.4589 \end{array}$	$\begin{array}{c} 5.3553e-04\\ 2.0263 \end{array}$	$\begin{array}{c} 1.3146e-04\\ 2.0373 \end{array}$	$\begin{array}{c} 3.2027e-05\\ 2.5131 \end{array}$	$\begin{array}{c} 5.6105e-06 \end{array}$

**Table 2:** $E_{\varepsilon ,\mu }^{N}$ and $R_{\varepsilon ,\mu }^{N}$ of Example (2) when $\mu =10^{-3}$ and various values of ε.

ε↓	N					
	64	128	256	512	1024	2048
$10^{-7}$	$\begin{array}{c} 2.0700e-02\\ 2.0102 \end{array}$	$\begin{array}{c} 5.1385e-03\\ 1.9970 \end{array}$	$\begin{array}{c} 1.2873e-03\\ 2.0007 \end{array}$	$\begin{array}{c} 3.2166e-04\\ 2.0003 \end{array}$	$\begin{array}{c} 8.0404e-05\\ 2.0001 \end{array}$	$\begin{array}{c} 2.0100e-05 \end{array}$
$10^{-8}$	$\begin{array}{c} 2.4871e-02\\ 2.1257 \end{array}$	$\begin{array}{c} 5.6988e-03\\ 2.0006 \end{array}$	$\begin{array}{c} 1.4241e-03\\ 2.0007 \end{array}$	$\begin{array}{c} 3.5584e-04\\ 2.0002 \end{array}$	$\begin{array}{c} 8.8949e-05\\ 2.0001 \end{array}$	$\begin{array}{c} 2.2237e-05 \end{array}$
$10^{-9}$	$\begin{array}{c} 3.4755e-01\\ 5.4416 \end{array}$	$\begin{array}{c} 7.9970e-03\\ 2.4515 \end{array}$	$\begin{array}{c} 1.4620e-03\\ 2.0197 \end{array}$	$\begin{array}{c} 3.6054e-04\\ 2.0008 \end{array}$	$\begin{array}{c} 9.0088e-05\\ 2.0001 \end{array}$	$\begin{array}{c} 2.2521e-05 \end{array}$

**Table 3:** $E_{\varepsilon ,\mu }^{N}$ and $R_{\varepsilon ,\mu }^{N}$ of Example (3) when $\mu =10^{-3}$ and various values of ε.

ε↓	N					
	64	128	256	512	1024	2048
$10^{-7}$	$\begin{array}{c} 2.2013e-02\\ 1.9818 \end{array}$	$\begin{array}{c} 5.5732e-03\\ 1.9840 \end{array}$	$\begin{array}{c} 1.4088e-03\\ 1.9947 \end{array}$	$\begin{array}{c} 3.5349e-04\\ 1.9987 \end{array}$	$\begin{array}{c} 8.3454e-05\\ 1.9548 \end{array}$	$\begin{array}{c} 2.2817e-05 \end{array}$
$10^{-8}$	$\begin{array}{c} 2.7250e-02\\ 2.1375 \end{array}$	$\begin{array}{c} 6.1934e-03\\ 1.9899 \end{array}$	$\begin{array}{c} 1.5592e-03\\ 1.9948 \end{array}$	$\begin{array}{c} 3.9122e-04\\ 2.0094 \end{array}$	$\begin{array}{c} 9.7167e-05\\ 2.6843 \end{array}$	$\begin{array}{c} 1.5117e-05 \end{array}$
$10^{-9}$	$\begin{array}{c} 5.0441e-02\\ 2.4066 \end{array}$	$\begin{array}{c} 9.5133e-03\\ 2.5800 \end{array}$	$\begin{array}{c} 1.5910e-03\\ 2.0526 \end{array}$	$\begin{array}{c} 3.8350e-04\\ 1.9382 \end{array}$	$\begin{array}{c} 1.0007e-04\\ 1.5019 \end{array}$	$\begin{array}{c} 3.5334e-05 \end{array}$

**Table 4:** $E_{\varepsilon ,\mu }^{N}$ and $R_{\varepsilon ,\mu }^{N}$ of Example (1) when $\mu =10^{-8}$ and various values of ε.

ε↓	N					
	64	128	256	512	1024	2048
$10^{-5}$	$\begin{array}{c} 3.5599e-03\\ 2.0325 \end{array}$	$\begin{array}{c} 8.7018e-04\\ 2.0038 \end{array}$	$\begin{array}{c} 2.1698e-04\\ 2.0023 \end{array}$	$\begin{array}{c} 5.4160e-05\\ 2.0005 \end{array}$	$\begin{array}{c} 1.3535e-05\\ 1.9999 \end{array}$	$\begin{array}{c} 3.3838e-06 \end{array}$
$10^{-6}$	$\begin{array}{c} 3.5601e-03\\ 2.0324 \end{array}$	$\begin{array}{c} 8.7025e-04\\ 2.0037 \end{array}$	$\begin{array}{c} 2.1700e-04\\ 2.0023 \end{array}$	$\begin{array}{c} 5.4165e-05\\ 2.0006 \end{array}$	$\begin{array}{c} 1.3536e-05\\ 2.0001 \end{array}$	$\begin{array}{c} 3.3837e-06 \end{array}$
$10^{-7}$	$\begin{array}{c} 3.5601e-03\\ 2.0324 \end{array}$	$\begin{array}{c} 8.7025e-04\\ 2.0037 \end{array}$	$\begin{array}{c} 2.1700e-04\\ 2.0023 \end{array}$	$\begin{array}{c} 5.4165e-05\\ 2.0006 \end{array}$	$\begin{array}{c} 1.3536e-05\\ 1.9998 \end{array}$	$\begin{array}{c} 3.3845e-06 \end{array}$
$10^{-8}$	$\begin{array}{c} 3.5600e-03\\ 2.0324 \end{array}$	$\begin{array}{c} 8.7021e-04\\ 2.0037 \end{array}$	$\begin{array}{c} 2.1699e-04\\ 2.0022 \end{array}$	$\begin{array}{c} 5.4163e-05\\ 2.0005 \end{array}$	$\begin{array}{c} 1.3536e-05\\ 2.0002 \end{array}$	$\begin{array}{c} 3.3836e-06 \end{array}$
$10^{-9}$	$\begin{array}{c} 3.5596e-03\\ 2.0325 \end{array}$	$\begin{array}{c} 8.7010e-04\\ 2.0038 \end{array}$	$\begin{array}{c} 2.1696e-04\\ 2.0022 \end{array}$	$\begin{array}{c} 5.4159e-05\\ 2.0003 \end{array}$	$\begin{array}{c} 1.3537e-05\\ 1.9883 \end{array}$	$\begin{array}{c} 3.4118e-06 \end{array}$
$10^{-10}$	$\begin{array}{c} 3.5582e-03\\ 2.0325 \end{array}$	$\begin{array}{c} 8.6976e-04\\ 2.0038 \end{array}$	$\begin{array}{c} 2.1687e-04\\ 2.100 \end{array}$	$\begin{array}{c} 5.4180e-05\\ 2.0018 \end{array}$	$\begin{array}{c} 1.3528e-05\\ 2.0001 \end{array}$	$\begin{array}{c} 3.3818e-06 \end{array}$

**Table 5:** $E_{\varepsilon ,\mu }^{N}$ and $R_{\varepsilon ,\mu }^{N}$ of Example (2) when $\mu =10^{-8}$ and various values of ε.

ε↓	N					
	64	128	256	512	1024	2048
$10^{-5}$	$\begin{array}{c} 9.6777e-03\\ 2.0324 \end{array}$	$\begin{array}{c} 2.3657e-03\\ 2.0037 \end{array}$	$\begin{array}{c} 5.8987e-04\\ 2.0022 \end{array}$	$\begin{array}{c} 1.4724e-04\\ 2.0006 \end{array}$	$\begin{array}{c} 3.6795e-05\\ 2.0001 \end{array}$	$\begin{array}{c} 9.1982e-06 \end{array}$
$10^{-6}$	$\begin{array}{c} 9.678e-03\\ 2.0325 \end{array}$	$\begin{array}{c} 2.3656e-03\\ 2.0037 \end{array}$	$\begin{array}{c} 5.8987e-04\\ 2.0022 \end{array}$	$\begin{array}{c} 1.4724e-04\\ 2.0005 \end{array}$	$\begin{array}{c} 3.6796e-05\\ 2.0001 \end{array}$	$\begin{array}{c} 9.1981e-06 \end{array}$
$10^{-7}$	$\begin{array}{c} 9.6778e-03\\ 2.0324 \end{array}$	$\begin{array}{c} 2.3657e-03\\ 2.0038 \end{array}$	$\begin{array}{c} 5.8988e-04\\ 2.0023 \end{array}$	$\begin{array}{c} 1.4724e-04\\ 2.0005 \end{array}$	$\begin{array}{c} 3.6796e-05\\ 2.0001 \end{array}$	$\begin{array}{c} 9.1982e-06 \end{array}$
$10^{-8}$	$\begin{array}{c} 9.6782e-03\\ 2.0324 \end{array}$	$\begin{array}{c} 2.3658e-03\\ 2.0038 \end{array}$	$\begin{array}{c} 5.8990e-04\\ 2.0022 \end{array}$	$\begin{array}{c} 1.4725e-04\\ 2.0006 \end{array}$	$\begin{array}{c} 3.6798e-05\\ 2.0001 \end{array}$	$\begin{array}{c} 9.1986e-06 \end{array}$
$10^{-9}$	$\begin{array}{c} 9.6793e-03\\ 2.0324 \end{array}$	$\begin{array}{c} 2.3661e-03\\ 2.0038 \end{array}$	$\begin{array}{c} 5.8998e-04\\ 2.0022 \end{array}$	$\begin{array}{c} 1.4727e-04\\ 2.0006 \end{array}$	$\begin{array}{c} 3.6802e-05\\ 2.0001 \end{array}$	$\begin{array}{c} 9.1998e-06 \end{array}$
$10^{-10}$	$\begin{array}{c} 9.6831e-03\\ 2.0324 \end{array}$	$\begin{array}{c} 2.3670e-03\\ 2.0038 \end{array}$	$\begin{array}{c} 5.9021e-04\\ 2.0022 \end{array}$	$\begin{array}{c} 1.4733e-04\\ 2.0006 \end{array}$	$\begin{array}{c} 3.6817e-05\\ 2.0001 \end{array}$	$\begin{array}{c} 9.2035e-06 \end{array}$

**Table 6:** $E_{\varepsilon ,\mu }^{N}$ and $R_{\varepsilon ,\mu }^{N}$ of Example (3) when $\mu =10^{-8}$ and various values of ε.

ε↓	N					
	64	128	256	512	1024	2048
$10^{-5}$	$\begin{array}{c} 1.0482e-02\\ 2.0134 \end{array}$	$\begin{array}{c} 2.5962e-03\\ 1.9974 \end{array}$	$\begin{array}{c} 6.5024e-04\\ 2.0010 \end{array}$	$\begin{array}{c} 1.6245e-04\\ 2.0002 \end{array}$	$\begin{array}{c} 4.0606e-05\\ 2.0021 \end{array}$	$\begin{array}{c} 1.0137e-05 \end{array}$
$10^{-6}$	$\begin{array}{c} 1.0482e-02\\ 2.0134 \end{array}$	$\begin{array}{c} 2.5962e-03\\ 1.9974 \end{array}$	$\begin{array}{c} 6.5024e-04\\ 2.0010 \end{array}$	$\begin{array}{c} 1.6245e-04\\ 2.0001 \end{array}$	$\begin{array}{c} 4.0610e-05\\ 1.9977 \end{array}$	$\begin{array}{c} 1.0169e-05 \end{array}$
$10^{-7}$	$\begin{array}{c} 1.0482e-02\\ 2.0134 \end{array}$	$\begin{array}{c} 2.5963e-03\\ 1.9974 \end{array}$	$\begin{array}{c} 6.5024e-04\\ 2.0010 \end{array}$	$\begin{array}{c} 1.6245e-04\\ 2.0003 \end{array}$	$\begin{array}{c} 4.0605e-05\\ 2.0073 \end{array}$	$\begin{array}{c} 1.0100e-05 \end{array}$
$10^{-8}$	$\begin{array}{c} 1.0483e-02\\ 2.0135 \end{array}$	$\begin{array}{c} 2.5963e-03\\ 1.9973 \end{array}$	$\begin{array}{c} 6.5027e-04\\ 2.0009 \end{array}$	$\begin{array}{c} 1.6247e-04\\ 2.0006 \end{array}$	$\begin{array}{c} 4.0601e-05\\ 1.969 \end{array}$	$\begin{array}{c} 1.0370e-05 \end{array}$
$10^{-9}$	$\begin{array}{c} 1.0484e-02\\ 2.0134 \end{array}$	$\begin{array}{c} 2.5967e-03\\ 1.9974 \end{array}$	$\begin{array}{c} 6.5035e-04\\ 2.0010 \end{array}$	$\begin{array}{c} 1.6247e-04\\ 2.0012 \end{array}$	$\begin{array}{c} 4.0584e-05\\ 1.9970 \end{array}$	$\begin{array}{c} 1.0167e-05 \end{array}$
$10^{-10}$	$\begin{array}{c} 1.0488e-02\\ 2.0135 \end{array}$	$\begin{array}{c} 2.5976e-03\\ 1.9973 \end{array}$	$\begin{array}{c} 6.5060e-04\\ 2.0003 \end{array}$	$\begin{array}{c} 1.6262e-04\\ 1.9831 \end{array}$	$\begin{array}{c} 4.1134e-05\\ 1.7958 \end{array}$	$\begin{array}{c} 1.1847e-05 \end{array}$

**Table 7:** Comparison of $E_{\varepsilon ,\mu }^{N}$ and $R_{\varepsilon ,\mu }^{N}$ for Example (1) when $\mu =10^{-8}$ and N=128.

	ε			
	$10^{-6}$	$10^{-8}$	$10^{-10}$	$10^{-12}$
Method in [22]	$\begin{array}{c} 9.0080e-03\\ 1.6351 \end{array}$	$\begin{array}{c} 9.0085e-03\\ 1.6351 \end{array}$	$\begin{array}{c} 9.0125e-03\\ 1.6351 \end{array}$	$\begin{array}{c} 9.0526e-03\\ 1.6350 \end{array}$
Present Method	$\begin{array}{c} 8.7025e-04\\ 2.0037 \end{array}$	$\begin{array}{c} 8.7021e-04\\ 2.0037 \end{array}$	$\begin{array}{c} 8.6976e-04\\ 2.0038 \end{array}$	$\begin{array}{c} 8.700e-04\\ 1.9967 \end{array}$

**Table 8:** Comparison of $E_{\varepsilon ,\mu }^{N}$ and $R_{\varepsilon ,\mu }^{N}$ for Example (2) when $\mu =10^{-3}$ and $\varepsilon =10^{-8}$.

	N				
	1000	2000	4000	8000	16000
Method in [31]	$\begin{array}{c} 1.390503e-02\\ 0.6985 \end{array}$	$\begin{array}{c} 8.568233e-03\\ 0.9123 \end{array}$	$\begin{array}{c} 4.552693e-03\\ 0.9085 \end{array}$	$\begin{array}{c} 2.425342e-03\\ 0.8971 \end{array}$	$\begin{array}{c} 1.302366e-03 \end{array}$
Present Method	$\begin{array}{c} 9.326971e-05\\ 2.00002 \end{array}$	$\begin{array}{c} 2.331708e-05\\ 1.99997 \end{array}$	$\begin{array}{c} 5.829395e-06\\ 1.9988 \end{array}$	$\begin{array}{c} 1.458533e-06\\ 1.9677 \end{array}$	$\begin{array}{c} 3.728777e-07 \end{array}$

**Table 9:** Comparison of $E_{\varepsilon ,\mu }^{N}$ and $R_{\varepsilon ,\mu }^{N}$ for Example (2) when $\mu =10^{-8}$ and $\varepsilon =10^{-6}$.

	N				
	64	128	256	512	1024
Method in [26]	$\begin{array}{c} 1.1567e-03\\ 1.9522 \end{array}$	$\begin{array}{c} 2.9890e-04\\ 1.9876 \end{array}$	$\begin{array}{c} 7.5369e-05\\ 1.9972 \end{array}$	$\begin{array}{c} 1.8879e-05\\ 1.9993 \end{array}$	$\begin{array}{c} 4.7220e-06 \end{array}$
Present Method	$\begin{array}{c} 9.678e-03\\ 2.0325 \end{array}$	$\begin{array}{c} 2.3656e-03\\ 2.0037 \end{array}$	$\begin{array}{c} 5.8987e-04\\ 2.0022 \end{array}$	$\begin{array}{c} 1.4724e-04\\ 2.0005 \end{array}$	$\begin{array}{c} 3.6796e-05 \end{array}$

## Conclusion

From the discussion in the above section it can be concluded that, the method is shown to: approximate the solution very well, be parameters uniform convergent of order two in the maximum norm, be more efficient than some methods in the existing literature and adaptable for computer implementation.

## CRediT authorship contribution statement

**Fellek Sabir Andisso:** Resources, Conceptualization, Methodology, Software, Investigation, Writing – original draft. **Gemechis File Duressa:** Methodology, Software, Investigation, Supervision, Validation, Writing – review & editing.

## Declaration of Competing Interest

The authors declare that they have no known competing financial interests or personal relationships that could have appeared to influence the work reported in this paper.

## Data Availability

The data that has been used is confidential.
